# Deciphering the therapeutic potential of trimetazidine in rheumatoid arthritis *via* targeting mi-RNA128a, TLR4 signaling pathway, and adenosine-induced FADD-microvesicular shedding: *In vivo* and *in silico* study

**DOI:** 10.3389/fphar.2024.1406939

**Published:** 2024-06-11

**Authors:** Enas Omran, Abdullah R. Alzahrani, Samar F. Ezzat, Ghada Ellithy, Marwa Tarek, Eman Khairy, Mohamed M. Ghit, Ahmed Elgeushy, Tahani Mohamed Ibrahim Al-Hazani, Ibrahim Abdel Aziz Ibrahim, Alaa Hisham Falemban, Ghazi A. Bamagous, Nasser A. Elhawary, Mariusz Jaremko, Essa M. Saied, Doaa I. Mohamed

**Affiliations:** ^1^ Department of Clinical Pharmacology and Therapeutics, Faculty of Medicine, Ain Shams University, Cairo, Egypt; ^2^ Department of Pharmacology and Toxicology, Faculty of Medicine, Umm Al-Qura University, Makkah, Saudi Arabia; ^3^ Department of Histology and Cell Biology, Faculty of Medicine, Ain Shams University, Cairo, Egypt; ^4^ Department of Medical Biochemistry and Molecular Biology, Faculty of Medicine, Ain Shams University, Cairo, Egypt; ^5^ Department of Clinical Biochemistry, Faculty of Medicine, University of Jeddah, Jeddah, Saudi Arabia; ^6^ Department of Rheumatology and Rehabilitation, Faculty of Medicine, Al-Azhar University, Cairo, Egypt; ^7^ Orthopedic Department, Faculty of Medicine, Alazhar University Hospitals, Cairo, Egypt; ^8^ Biology Department, College of Science and Humanities, Prince Sattam Bin Abdulaziz University, Al-Kharj, Saudi Arabia; ^9^ Department of Medical Genetics, College of Medicine, Umm Al-Qura University, Mecca, Saudi Arabia; ^10^ Smart-Health Initiative and Red Sea Research Center, Division of Biological and Environmental Sciences and Engineering, King Abdullah University of Science and Technology, Thuwal, Saudi Arabia; ^11^ Chemistry Department, Faculty of Science, Suez Canal University, Ismailia, Egypt; ^12^ Institute for Chemistry, Humboldt Universität zu Berlin, Berlin, Germany

**Keywords:** rheumatoid arthritis, mi-RNA128a, FADD-microvesicular shedding, TLR4, adenosine level, trimetazidine, immunological and inflammatory markers, histopathological analysis

## Abstract

Rheumatoid arthritis (RA) is a debilitating autoimmune condition characterized by chronic synovitis, joint damage, and inflammation, leading to impaired joint functionality. Existing RA treatments, although effective to some extent, are not without side effects, prompting a search for more potent therapies. Recent research has revealed the critical role of FAS-associated death domain protein (FADD) microvesicular shedding in RA pathogenesis, expanding its scope beyond apoptosis to include inflammatory and immune pathways. This study aimed to investigate the intricate relationship between mi-RNA 128a, autoimmune and inflammatory pathways, and adenosine levels in modulating FADD expression and microvesicular shedding in a Freund’s complete adjuvant (FCA) induced RA rat model and further explore the antirheumatoid potency of trimetazidine (TMZ). The FCA treated model exhibited significantly elevated levels of serum fibrogenic, inflammatory, immunological and rheumatological diagnostic markers, confirming successful RA induction. Our results revealed that the FCA-induced RA model showed a significant reduction in the expression of FADD in paw tissue and increased microvesicular FADD shedding in synovial fluid, which was attributed to the significant increase in the expression of the epigenetic miRNA 128a gene in addition to the downregulation of adenosine levels. These findings were further supported by the significant activation of the TLR4/MYD88 pathway and its downstream inflammatory IkB/NFB markers. Interestingly, TMZ administration significantly improved, with a potency similar to methotrexate (MTX), the deterioration effect of FCA treatment, as evidenced by a significant attenuation of fibrogenic, inflammatory, immunological, and rheumatological markers. Our investigations indicated that TMZ uniquely acted by targeting epigenetic miRNA128a expression and elevating adenosine levels in paw tissue, leading to increased expression of FADD of paw tissue and mitigated FADD microvesicular shedding in synovial fluid. Furthermore, the group treated with TMZ showed significant downregulation of TLR4/MYD88 and their downstream TRAF6, IRAK and NF-kB. Together, our study unveils the significant potential of TMZ as an antirheumatoid candidate, offering anti-inflammatory effects through various mechanisms, including modulation of the FADD-epigenetic regulator mi-RNA 128a, adenosine levels, and the TLR4 signaling pathway in joint tissue, but also attenuation of FADD microvesicular shedding in synovial fluid. These findings further highlight the synergistic administration of TMZ and MTX as a potential approach to reduce adverse effects of MTX while improving therapeutic efficacy.

## Graphical Abstract

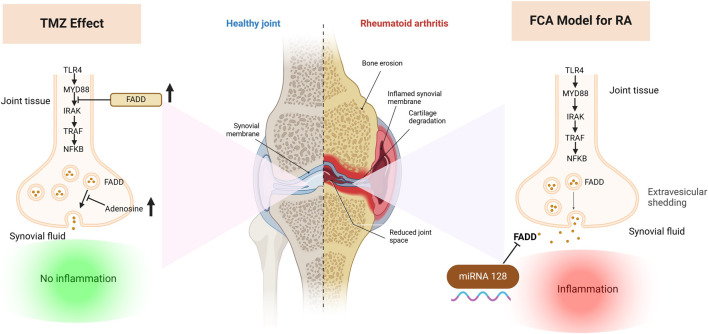

## 1 Introduction

Rheumatoid arthritis (RA) is a long-lasting autoimmune disease that damages the joints by causing persistent inflammation of the synovial membrane, cell infiltration, and vasospasm (Smolen et al., 2016). The exact mechanisms behind the development of RA remain elusive. However, a substantial body of research has consistently demonstrated the involvement of several elements in the etiology of this disease, including environmental influences, infections, genetic predisposition, and inflammatory and autoimmune processes (Radu and Bungau, 2021). Synovial inflammatory regulation in RA involves a complex interplay of cytokines, chemokines, Toll-like receptors (TLR), tumor necrosis factor-alpha (TNF-α), and nuclear factor kappa B (NF-κB), alongside numerous other inflammatory mediators (Schaper et al., 2015). TLR4 activation through interaction with MYD88, a key gene involved in myeloid differentiation (Ospelt et al., 2008a), triggers multiple inflammatory responses, initiating a cascade of signals, including phosphorylation of tumor necrosis factor receptor-associated factor 6 (TRAF6) by interleukin-1 receptor-associated kinase 1 (IRAK1) (Scarneo et al., 2020). Subsequently, this process upregulates NF-κB expression, which in turn elevates the expression of inflammatory genes such as IL-6, vascular endothelial growth factor (VEGF), and matrix metalloproteinases (MMPs) (Barton and Medzhitov, 2003; Park and Hong, 2016). NF-κB activation is also initiated through the phosphorylation of IκB (inhibitor of NF-κB) by IκB kinase (IKK), enabling NF-κB to migrate from the cytoplasm to the nucleus, where it regulates the expression of inflammatory genes (Christian et al., 2016; Su et al., 2018).

The FAS-associated death domain protein (FADD) plays a vital role in regulating inflammation through its ability to inhibit the TLR4 signaling pathway (Zhande et al., 2007). To achieve this, it binds to the key responder gene for MYD88, therefore suppressing NFkB signaling. Dysregulation of intracellular FADD levels can lead to defective apoptosis and increased proliferation of fibroblast-like synoviocyte (FLS) proliferation (Ma et al., 2004). Extracellular vesicles (EV) are pivotal mediators of cell-to-cell communication within synovial joints, particularly in the context of RA pathogenesis (Alghamdi et al., 2022). These electric vehicles encompass diverse structures including microvesicles that are formed by direct growth and shedding from the cell membrane (Akers et al., 2013; Raposo and Stoorvogel, 2013). Several evidences suggest that EVs contribute significantly to RA progression by promoting the synthesis of inflammatory mediators that act as key players in the pathogenesis of the disease, including IL-6, matrix MMPs, and VEGF (Reich et al., 2011). In joints of RA, EV mediate, through an ATP-dependent mechanism, the elimination of FADD leading to a significant depletion of this anti-inflammatory agent in the tissue, exacerbating joint inflammation (Tourneur and Chiocchia, 2010). In this sense, adenosine has emerged as a key regulator for this process, as it inhibits the release of FADD-containing microvesicles into the synovial fluid (Tourneur et al., 2008; Vilmont et al., 2012). Therefore, increasing adenosine levels has emerged as a potential strategy to modulate RA progression by attenuating microvascular shedding of FADD in joint tissues. MiRNAs are short noncoding RNA molecules (18–25 nucleotides) that play key roles in the regulation of various biological processes, and their dysregulation has been linked to several human diseases (Bartel, 2009). Recently, epigenetic modifications have gained attention as potential mechanisms contributing to RA pathogenesis (Menigatti et al., 2013). An interesting study by Yamada *et al.* revealed that miR-128a exerts its regulatory influence on the apoptosis pathway by modulating the expression of FADD. In T cell leukemia cells resistant to Fas-induced apoptosis, the demethylation process leads to an increase in miR-128a levels that bind directly to the 3-UTR of FADD, causing delayed onset of apoptosis (Yamada et al., 2014). These findings indicate that miRNA-128a could be an epigenetic regulator of the FADD-associated pathway, and further highlight the possibility that miRNA-128a is associated with RA diseases.

To date, there is no potential drug for complete treatment of RA disease. Methotrexate (MTX) is one of the disease-modifying antirheumatic drugs that serves as a first-line treatment for RA (Visser et al., 2009). The antirheumatoid activity of MTX is attributed to its ability to target the adenosine signaling pathway in RA by elevating its intracellular and extracellular levels by preventing its breakdown to inosine. Additionally, MTX decreases the production of proinflammatory cytokines like NFkB (Zhao et al., 2022). Despite its efficacy, MTX can cause various adverse effects. MTX can interfere with folate metabolism resulting in gastrointestinal side effects, elevated serum transaminase levels, bone marrow suppression, hair loss, and even hepatic and pulmonary fibrosis (Friedman and Cronstein, 2019). Considering the increasing prevalence of rheumatoid arthritis around the world and the lack of effective drugs that cause fewer side effects, the development of drugs with greater efficacy is of utmost importance. Trimetazidine (TMZ), a commonly used drug in the treatment of coronary artery disease, increases conventional antianginal therapies by modulating cardiac metabolism (Rakočević et al., 2023). Its antioxidant characteristics mitigate inflammatory responses by inhibiting the production of cytokines such as TNF-α, NFKB, and IL-6 in macrophages (Dézsi, 2016). TMZ also exerts its cytoprotective effect by regulating adenosine levels by targeting the final enzyme in fatty acid β-oxidation (3-ketoacyl coenzyme A thiolase). This alteration redirects energy utilization, decreasing fatty acid oxidation while enhancing glucose oxidation (Luo et al., 2021). Furthermore, TMZ boasts a distinct safety profile characterized by minimal adverse effects and overall good tolerability. A systematic review and meta-analysis evaluating trimetazidine usage revealed low numbers of observed adverse events and dropout rates (Ciapponi et al., 2005). These experienced side effects are typically mild and resolve upon discontinuation of the drug. The ability of TMZ to modulate adenosine levels together with its pronounced antioxidant and anti-inflammatory activities highlight its therapeutic application as an anti-rheumatoid drug.

Based on the facts mentioned above, the present study aimed to explore the molecular epigenetic regulation of FADD expressions by targeting miRNA 128a, which could be a unique diagnostic biomarker in RA and a potential target for therapeutic intervention. Additionally, the possible connection between FADD micro vesicular shedding and adenosine levels in FCA-induced RA was also investigated. We further aimed to unveil the possible antirheumatoid effect of TMZ, either alone or in synergetic combination with MTX, to target the proposed pathway through physical, biochemical, molecular modelling, histological analysis.

## 2 Results

### 2.1 TMZ attenuated changes in paw volume, ankle diameter, and body weight in FCA-induced RA

**FIGURE 1:**
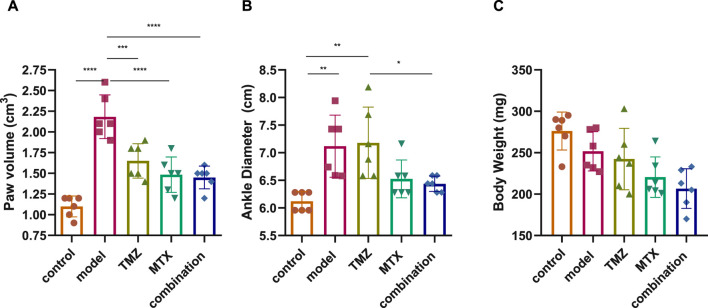
Effect of trimetazidine (TMZ), methotrexate (MTX) and their combination on body paw volume **(A)**, ankle diameter **(B)** and body weight **(C)** of FCA-induced RA rats compared to the control model. Data are presented as mean ± SE (n = 6). Multiple comparisons were analyzed by one-way ANOVA followed by Tukey’s multiple comparison test. Differences between groups were considered significant when *p* < 0.05 (^∗^ *p* < 0.05; ^∗∗^ *p* < 0.01; ^∗∗∗^ *p* < 0.001).

We initially evaluated the severity of RA by scoring the volume of the paw, the diameter of the ankle, and the body weight of the FCA-induced RA model and the drug-treated groups. The evaluations were carried out compared to control rats over a period of 0, 4, 7, 10, 14, 18, and 21 days, and the significance between the groups was considered on the 21st day to determine the net effect (Supplementary Figure S1). Our analysis revealed that the FCA treated group exhibits a significant (*p* < 0.001) increase in paw volume and ankle diameter, while there was a nonsignificant decrease in body weight compared to the control group (Figure 1). These findings suggest that the administration of FCA induced joint inflammation characterized by edema and swelling, as evidenced by the increase in the volume of the paw and the diameter of the ankle, which are typical features of rheumatoid arthritis. The observed nonsignificant decrease in body weight of the FCA-treated group could be attributed to the FCA-induced inflammatory response. Consistent with previous reports, our findings indicate that FCA is a suitable model for RA induction. Next, we evaluated the impact of TMZ, alone or in combination with MTX, on improving the examined features in the FCA-treated model. As shown in Figure 1, in accordance with previous studies, MTX treatment showed a significant (*p* < 0.001) alleviation in paw volume and ankle diameter compared to the FCA-induced RA model, while the reduction in body weight was not significant. The nonsignificant decrease in weight could also be attributed to the convenient access to food which was provided inside the cage. Similarly, the TMZ-treated group exhibited a significant (*p* < 0.001) mitigation in paw volume compared to the FCA-induced RA model. However, the reduction in ankle volume and body weight was not significant compared to the FCA-treated group. Interestingly, the synergetic administration of TMZ and MTX demonstrated the most effectiveness toward the examined RA features. Our results showed that the combination group has a significant (*p* < 0.001) reduction (*p* < 0.001) in paw volume and ankle diameter, compared to the FCA-induced RA group, while the substantial decrease in body weight was not significant (Figure 1). These findings indicate that TMZ exhibits anti-inflammatory potential toward RA by alleviating signs of inflammation, such as edema and swelling, with a notable preference for the combination treatment group.

### 2.2 TMZ significantly elevated FADD expression in joint tissue by modulating miRNA-128a expression and adenosine levels in FCA-induced RA

**FIGURE 2:**
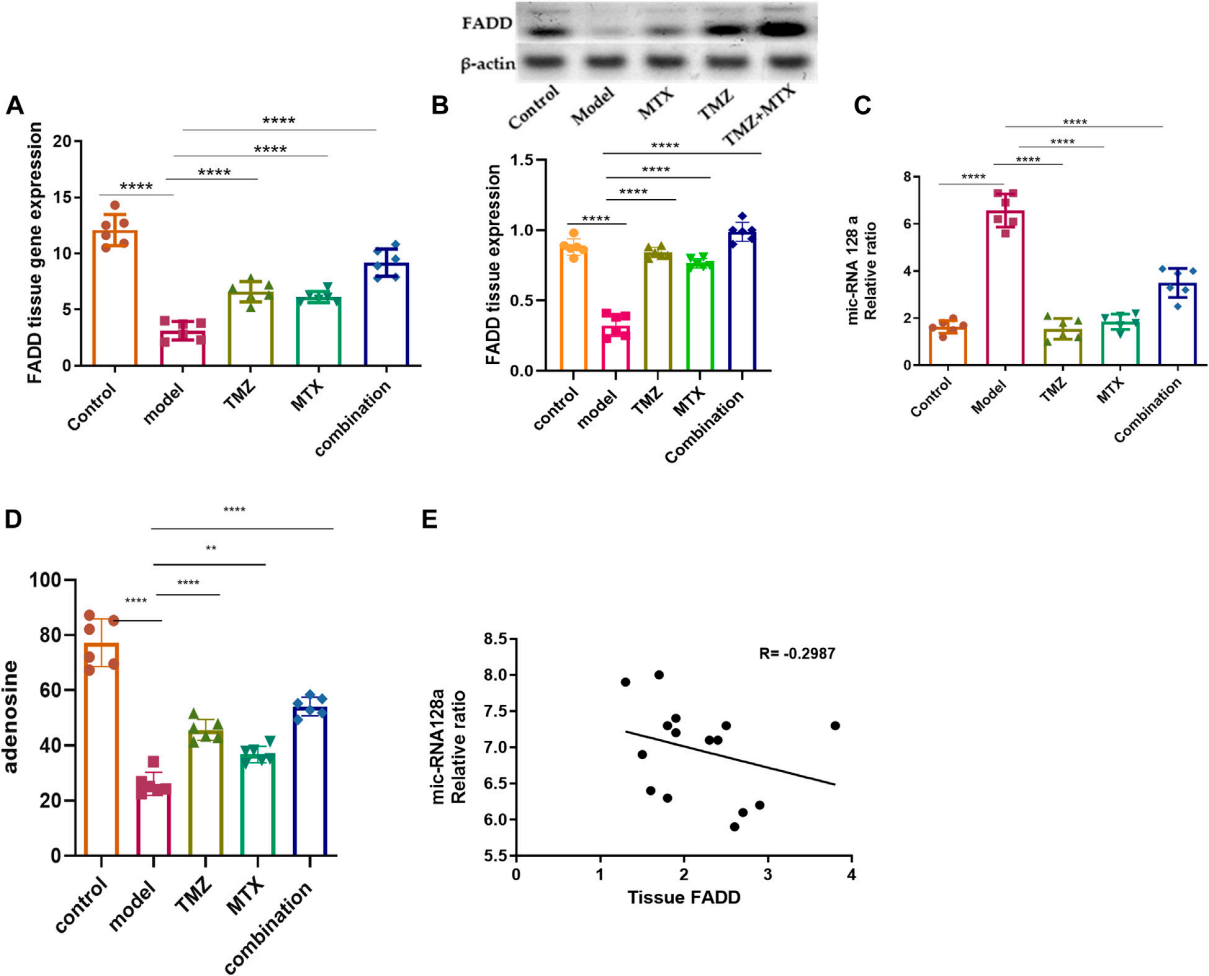
Effect of trimetazidine (TMZ), methotrexate (MTX), and their combination on the expression levels of tissue FADD **(A, B)**, mi-RNA128a **(C)**, and adenosine **(D)** of FCA-induced RA rats compared to the control model. The correlation analysis between joint tissue FADD and mi-RNA128a **(E)**. Data are presented as mean ± SE (n = 6). Multiple comparisons were analyzed by one-way ANOVA followed by Tukey’s multiple comparison test. Differences between groups were considered significant when *p* < 0.05 (^∗^ *p* < 0.05; ^∗∗^ *p* < 0.01; ^∗∗∗^ *p* < 0.001).

The role of FADD in RA extends beyond its involvement in apoptosis and encompasses its participation in inflammatory and immune pathways. Its dysregulation can affect the persistence of inflammatory cells, the release of pro-inflammatory triggers, and the viability of crucial cell types in synovial tissue. Together, these factors contribute to the pathogenesis and progression of RA. In this regard, we examine the antirheumatoid effect of TMZ by examining its ability to modulate FADD expression levels in the RA model. As shown in Figure 2A, FCA administration showed significant attenuation (*p* < 0.001) in FADD expression levels in joint tissue, compared to the control group. The observed downregulation in FADD expression, in agreement with previous studies, is associated with the trigger of both apoptotic and pro-inflammatory pathways that impact the progression of RA. Our findings further indicate the pronounced influence of FCA on the downregulation of FADD, supporting the hypothesis that FCA is appropriate for the development of the RA model. Interestingly, the administration of TMZ or MTX demonstrated a significant (*p* < 0.001) upregulation in the expression levels in paw tissue, compared to the FCA-treated group. However, the MTX-treated group exhibited a decrease in tissue FADD expression compared to the control group. The treated group with synergetic treatment of TMZ and MTX demonstrated a more dramatic and significant elevation (*p* < 0.001) in FADD protein expression compared to the FCA treated model. To further confirm these findings, we have performed a Western plot analysis to quantify the expression of the FADD protein in the paw tissues of different treated RA groups. As shown in Figure 2B, the analysis supported our PCR analysis in the different groups. Compared to the control group, the FCA-induced RA model group demonstrated attenuated expression of tissue FADD protein, while expression was dramatically restored in the drug-treated groups. Again, the TMZ- and MTX-treated groups exhibited a significant increase in FADD expression compared to the FCA-treated model. Furthermore, the MTX + TMZ combination treated group showed the highest expression levels of FADD protein. FADD has been established as a regulator of inflammation due to its ability to suppress the expression of various inflammatory cytokines. Our results suggest that TMZ has potential as an antirheumatoid drug that can affect the expression of the FADD protein in joint tissue and modulate RA disease. Synergetic treatment appears to have a more pronounced effect on restoring FADD levels, potentially contributing to its enhanced therapeutic efficacy.

Next, our investigations were directed to reveal the molecular mechanism of TMZ to modulate the expression of FADD in RA paw tissue. In this regard, we explored the epigenetic regulation of the FADD protein in the RA model. Previous evidence has indicated that miR-128a plays a crucial role in modulating Fas-mediated apoptosis in human T cell leukemia by directly targeting FADD, suggesting that mi-RNA128a could be an epigenetic regulator of the FADD protein in RA. Toward this, our aim was to explore whether the mi-RNA 128a gene is involved in the progression of RA disease and to reveal whether its expression could impact subsequent expression of FADD, thus influencing the inflammatory pathways associated with RA. As depicted in Figure 2C, our results revealed that FCA administration significantly (*p* < 0.001) enhanced the expression of mi-RNA 128a expression in paw tissue compared to the control group. Consistent with previous reports, our findings indicate the pronounced influence of FCA on mi-RNA128a upregulation, shedding light on the potential link between FADD and mi-RNA 128a in the pathogenesis of RA. To further explore this hypothesis, we conducted a statistical analysis using Pearson’s correlation coefficient to explore the correlation between tissue FADD protein expression and its suspected epigenetic regulator mi-RNA 128a. As shown in Figure 2D, our results revealed a significant negative correlation between tissue FADD protein and tissue mi-RNA 128a (R = −0.2987, *p* > 0.05). These results indicate the possible unique relation between the expression of the FADD protein and mi-RNA 128a in paw tissue and further underscore the potential of mi-RNA 128a as a unique and novel therapeutic target and prognostic marker in RA patients. Next, we explored whether the antirheumatoid activity of TMZ is associated with its ability to target mi-RNA 128a by examining the expression of mi-RNA 128a in paw tissue of drug-treated groups. Aligning with their effect on FADD expression, the results showed that TMZ or MTX administration significantly attenuated (*p* < 0.001) the expression of mi-RNA 128a expression in paw tissue as compared to the FCA treated model. Similarly, synergetic treatment of both TMZ and MTX showed a more dramatic and significant decrease (*p* < 0.001) in mi-RNA 128a expression compared to the FCA-treated group. Together, our results provide, to our knowledge, the first evidence for the involvement of miRNA 128a in the progression of RA and suggest its potential as a unique therapeutic target for the treatment of RA by epigenetically modulating FADD expression in joint tissue and thereby the subsequent inflammatory pathways associated with RA. Furthermore, our analysis revealed that TMZ exhibits substantial antirheumatoid activity by targeting mi-RNA 128a expression in paw tissue, with preference for its combination with MTX.

To gain further insight into the ability of TMZ to modulate the expression of FADD in RA paw tissue, our objective was to explore whether the potency of TMZ to regulate the expression of FADD in RA paw tissue is associated with its ability to modulate adenosine levels. Adenosine has the capacity to downregulate the NF-κB pathway by suppressing microvesicular shedding of FADD, primarily through its impact on intracellular Ca^2+^ and cAMP levels, which results in inhibition of intracellular Ca^2+^ release. Furthermore, it plays a role in mitigating the pain and inflammation associated with the acute inflammatory phase of RA. Toward this end, we have evaluated the levels of adenosine in the paw tissue of the FCA-treated model and drug-treated groups. As shown in Figure 2D, the FCA-treated group showed a significant reduction (*p* < 0.001) in paw tissue adenosine levels compared to the control group. These results imply the notable influence of FCA treatment on tissue adenosine levels, prompting intriguing inquiries about the possible involvement of adenosine dysregulation in FCA-induced RA. On the other hand, administration of TMZ or MTX demonstrated a substantial elevation (*p* < 0.001) in tissue adenosine levels, compared to the FCA-treated group. Interestingly, combined treatment with TMZ and MTX showed the most significant increase (*p* < 0.001) in tissue adenosine levels compared to the FCA-induced RA model. The observed elevation of adenosine levels demonstrates robust anti-inflammatory effects through distinct pathways, including inhibition of cell apoptosis, mitigation of tissue inflammation, and modulation of FADD microvesicular shedding, which constitutes the central element of our presented antirheumatoid pathway. Together, these findings indicate that the antirheumatoid activity of TMZ is associated with alterations in tissue adenosine levels that play a vital role in modulating FADD expression in joint tissue and mitigating microvesicular shedding. Furthermore, our results reveal that the combination of TMZ and MTX appears to have a synergistic effect in increasing adenosine levels in the context of RA.

### 2.3 TMZ mitigated TLR4-induced MYD88 activation and its downstream TRAF6 and IRAK in FCA-induced RA

**FIGURE 3:**
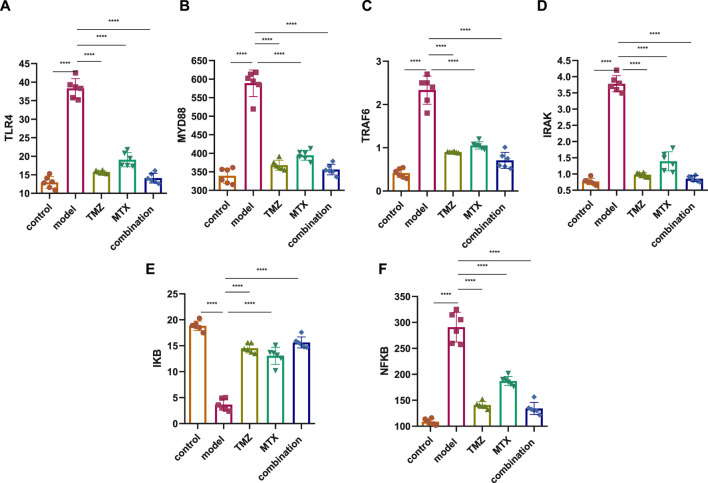
Effect of trimetazidine (TMZ), methotrexate (MTX), and their combination on the expression of TLR4 **(A)**, MYD88 **(B)**, TRAF6 **(C)**, IRAK **(D)**, IKB **(E)** and NFKB **(F)** proteins in the paw tissue of FCA-induced RA compared to the control model. Data are presented as mean ± SE (n = 6). Multiple comparisons were analyzed by one-way ANOVA followed by Tukey’s multiple comparison test. Differences between groups were considered significant when *p* < 0.05 (^∗^ *p* < 0.05; ^∗∗^ *p* < 0.01; ^∗∗∗^ *p* < 0.001).

Several studies demonstrated an increase in TLR4 expression on the cell membrane of RA cells that contributes to the pathogenesis of RA through the MDY88/TRAF6/IRAK pathway, suggesting the TLR4 pathway as a therapeutic target to attenuate the progression of RA. Toward this end, we have assessed the therapeutic potential of TMZ’s synergistic administration with MTX on the RA model by examining the expression of TLR4 and MYD88, but also its downstream level of TRAF6 and IRAK in the paw tissue of the FCA-induced RA model. As shown in Figure 3, the FCA-treated group exhibited a significant (*p* < 0.001) upregulation in the expression of the TLR4 and MYD88 level, compared to the control group. These results indicate that FCA administration successfully triggered the inflammatory process in the RA model. Aligning with these findings, the FCA-treated group further exhibited a significant (*p* < 0.001) increase (*p* < 0.001) in the expression levels of the downstream inflammatory biomarkers TRAF6 and IRAK, compared to the control group. MyD88 binds to IRAK, initiating subsequent phosphorylation processes that trigger the activation of TNF and TRAF6, thus amplifying inflammation. Our findings indicate that the observed elevation in IRAK and TRAF6 expression in the FCA-induced RA model is associated with the notable increase in the expression of MYD88 and TLR4, supporting their association with the inflammatory processes observed in the RA model and further affirm the inflammatory effect of FCA treatment. Next, we examine the impact of TMZ administration, alone or in synergetic combination with MTX, on modulating the expression levels of the examined proteins. As shown in Figure 3, administration of TMZ or MTX significantly attenuated the expression levels of TLR4 and MYD88 in paw tissues, compared to the FCA-induced RA model. The observed mitigation in TLR4 expression leads to decreased invasion and migration of fibrocyte-like synoviocytes, accompanied by a regression of inflammation, as indicated by suppression of MyD88 expression. Interestingly, the synergistic combination of TMZ and MTX demonstrated the most potent efficacy (*p* < 0.001) in reinstating TLR4 and MYD88 levels in the RA model. These results indicate that the antirheumatoid activity of TMZ could be associated with its ability to substantially target the TLR4/MYD88 pathway. Consistent with these findings, our results also showed that TMZ treatment, alone or in combination with MTX, significantly (*p* < 0.001) decreased the expression levels of downstream inflammatory biomarkers TRAF6 and IRAK in paw tissues, compared to the FCA-induced RA model (Figure 3). Similarly, synergetic administration of TMZ and MTX showed the most notable and significant (*p* < 0.001) attenuation (*p* < 0.001) in protein expression levels examined, compared to the FCA-induced RA model. These findings further support that the antirheumatoid activity of TMZ could be attributed to its potential to target the inflammatory TRAF6/IRAK pathway in the paw tissues of the RA model, with a preference effect for combination treatment with MTX. Together, our findings suggest that TMZ has substantial activity to attenuate the progression of RA by targeting TLR4/MYD88, which consequently leads to suppression of the associated downstream TRAF6/IRAK inflammatory pathway, suggesting its therapeutic potential as an antirhomboid drug. Furthermore, our results highlight the therapeutic application of TMZ in combination with MTX to reduce side effects related to MTX in RA treatment, but also to extensively mitigate inflammatory symptoms associated with RA.

### 2.4 TMZ moderated NF-κB expression through upregulation of IkB in FCA-induced RA

IkB is a complex enzyme crucial for cellular responses to inflammation, particularly in the regulation of lymphocytes and plays a pivotal role downstream of NF-κB signal transduction pathway. Toward this, we next aim to gain insight into the role of IkB/NF-κB pathway in RA by assessing its expressions in the paw tissue of FCA-induced RA. As shown in Figure 3, the FCA treated group demonstrated a significant (*p* < 0.001) decrease (*p* < 0.001) in IkB protein expression and a substantial (*p* < 0.001) upregulation in the expression of NF-κB protein, as compared to the control group. These findings indicate that decreased expression of IkB could be involved in the development or progression of RA, potentially contributing to the inflammatory processes observed in the FCA-induced RA model. Furthermore, these results underscore the pivotal role of the IkB/NF-κB pathway in the pathogenesis of RA, providing valuable information for potential therapeutic interventions targeting this pathway in the management of the disease. To further explore whether the antirheumatoid effect of TMZ, alone or in combination with MTX, is associated with the IkB/NF-κB pathway, the expression of these proteins was evaluated in the drug-treated groups. As shown in Figure 3, administration of MTX or TMZ significantly (*p* < 0.001) mitigated the expression of IkB and NF-κB proteins in the paw tissues of the FCA-treated model. Interestingly, the combination treatment of TMZ and MTX demonstrated the most significant (*p* < 0.001) effect (*p* < 0.001) on the expression of IkB and NF-κB proteins, compared to the RA model. These findings further confirm the anti-rheumatoid activity of TMZ in the RA model via suppression of IkB expression, which subsequently prevents activation of NF-κB, ultimately leading to the mitigation of joint arthritis. In particular, the combination treatment group exhibits a particularly favorable outcome in this regard, suggesting the therapeutic application of TMZ as a dose-sparing antirheumatoid drug.

### 2.5 TMZ significantly reduced the expression levels of serum rheumatological biomarkers in FCA-induced RA

**FIGURE 4:**
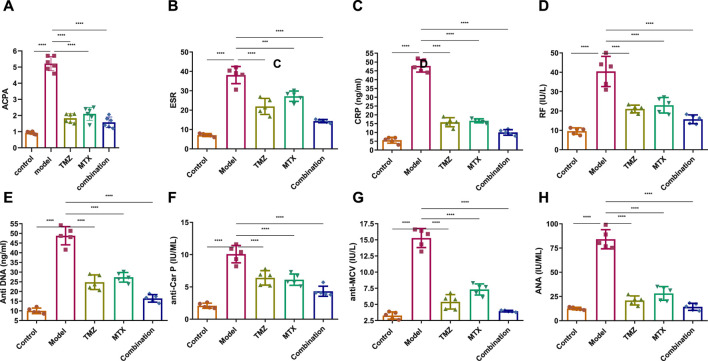
Effect of trimetazidine (TMZ), methotrexate (MTX), and their combination on the expression of the serum rheumatological biomarkers ACPA **(A)**, ESR **(B)**, CRP **(C)**, RF **(D)** and the serum immunological antibodies anti-DNA **(E)**, anti-CarP **(F)**, anti-MCV **(G)** and ANA **(H)** proteins in FCA-induced RA compared to the control model. Data are presented as mean ± SE (n = 6). Multiple comparisons were analyzed by one-way ANOVA followed by Tukey’s multiple comparison test. Differences between groups were considered significant when *p* < 0.05 (^∗^ *p* < 0.05; ^∗∗^ *p* < 0.01; ^∗∗∗^ *p* < 0.001).

To gain insight into the involvement of rheumatological biomarkers in the course and severity of RA, we evaluated the expression of a set of known serum diagnostic markers of rheumatoid arthritis, mainly ACPA, ESR, CRP and RF, which are sensitive but not specific markers for RA. As shown in Figure 4A–C, the FCA-treated group showed a significant (*p* < 0.001) increase (*p* < 0.001) in the expression of serum ACPA, ESR, CRP and RF proteins, compared to the control group. These results indicate that FCA administration successfully performed the induction of the RA model, as indicated by the elevation of the serum biomarkers examined. To further explore the antirheumatoid efficacy of TMZ, we evaluated the expression of these rheumatological biomarkers in the serum of drug-treated groups. Treatment of FCA-induced RA rats with MTX significantly (*p* < 0.001) attenuated the expression level of serum ACPA, ESR, CRP, and RF proteins (Figure 4). Similarly, the TMZ-treated group showed a significant (*p* < 0.001) reduction (*p* < 0.001) in the expression level of the examined proteins, compared to the FCA-treated group. Consistent with our presented findings, synergetic treatment of both TMZ and MTX demonstrated the most significant impact (*p* < 0.001) on the expression of examined rheumatological biomarkers, compared to the FCA-treated group. Substantial attenuation of sensitive rheumatological biomarkers in the TMZ-treated group underscores the potential therapeutic efficacy of TMZ. Additionally, these findings indicate that the antirheumatoid effect of TMZ may be associated with its ability to attenuate the inflammatory response associated with RA, with a preference effect toward synergetic treatment.

### 2.6 TMZ significantly attenuated the expression of serum immunological antibodies in FCA-induced RA

To gain a comprehensive understanding of the immunological etiology of RA, we evaluated the expression levels of serum immunological antibodies, including anti-dsDNA, anti-CarP, anti-MCV, and ANA. These markers, except the ANA antibody, have been established as reliable predictors and specific diagnostic indicators for RA. ANA functions as a sensitive, yet nonspecific indicator for RA. Our investigations revealed that FCA treatment significantly elevated (*p* < 0.001) the expression levels of serum anti-dsDNA, anti-CarP, anti-MCV, and ANA, compared to the control group (Figures 4E–H). These results indicate that FCA treatment has a significant impact on these specific markers, suggesting a potential association with the development or exacerbation of autoimmune responses in the RA model. Next, we assessed the effect of TMZ treatment on the expression of these serum biomarkers. As shown in Figure 4EH, both the TNZ and MTX treated groups showed significant and substantial (*p* < 0.001) attenuation in the expression levels of serum anti-dsDNA, anti-CarP, anti-MCV, and ANA proteins, compared to the FCA-induced RA model. These results highlight the synergistic potential of TMZ in influencing autoimmune responses in the RA model. Interestingly, the concurrent administration of TMZ and MTX exhibited the most substantial impact on the biomarkers examined, suggesting the therapeutic benefit of synergistic administration of TMZ as a dose-saving drug to reduce adverse effects associated with MTX in RA treatment. In general, our investigations indicate that the anti-rhmatoid activity of TMZ might be related to its immunomodulatory activity in RA.

### 2.7 TMZ significantly ameliorated the expression of serum fibrogenic and inflammatory biomarkers in FCA-induced RA

**FIGURE 5:**
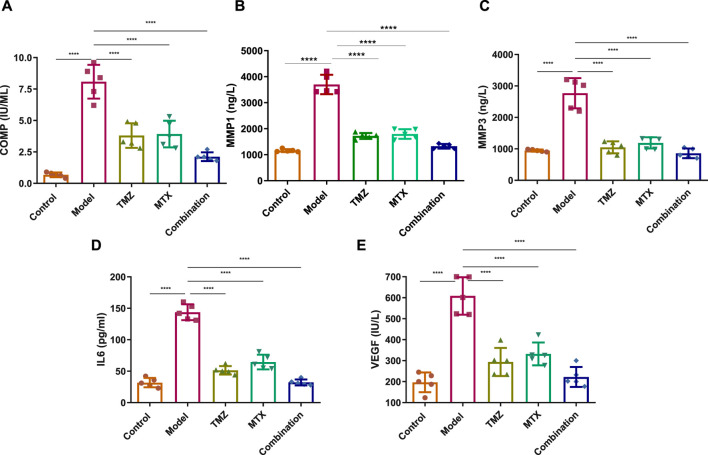
Effect of trimetazidine (TMZ), methotrexate (MTX), and their combination on the expression levels of serum fibrogenic COMP **(A)**, MMP-1 **(B)**, MMP-3 **(C)**, and inflammatory IL-6 **(D)** and VEGF **(E)** biomarkers in FCA-induced RA compared to the control model. Data are presented as mean ± SE (n = 6). Multiple comparisons were analyzed by one-way ANOVA followed by Tukey’s multiple comparison test. Differences between groups were considered significant when *p* < 0.05 (^∗^ *p* < 0.05; ^∗∗^ *p* < 0.01; ^∗∗∗^ *p* < 0.001).

To examine the extent of joint damage and the progression of RA disease, we assessed serum levels of a set of fibrogenic markers including cartilage oligomeric matrix protein (COMP) and matrix metalloproteinases (MMP1 and MMP3). As shown in Figure 5, the FCA treated group showed a statistically significant elevation (*p* < 0.001) in serum expression levels of COMP, MMP1 and MMP3, compared to the control group. These results indicate that FCA effectively induced RA in the experimental group, as the examined markers serve as indicators for both the level of disease and the degree of articular cartilage damage. We further examined the antirhumatoid activity of TMZ by evaluating the expression of these serum proteins in the drug-treated groups (Figure 5). Consistent with our previous findings, our results showed that TMZ or MTX administration, alone or in combination, significantly diminished (*p* < 0.001) the expression of serum levels of COMP, MMP1, and MMP3, compared to the FCA-induced RA model. Notably, within all treatment groups, synergetic administration of TMZ and MTX demonstrated the most pronounced effect on the biomarkers examined. These results offer strong evidence for the antirheumatoid activity of TMZ by targeting serum fibrogenic biomarkers, highlighting the therapeutic potential of TMZ in mitigating joint destruction and advancement of RA.

To gain further insight into the role of inflammatory pathways in TMZ antirheumatoid activity, our objective was to explore the expression levels of the serum IL-6 and VEGF protein in FCA-induced RA and drug-treated models. As shown in Figure 5, the FCA-treated group exhibited a significant elevation (*p* < 0.001) in the expression levels of serum IL-6 and VEGF, compared to the control group. Serum IL-6 and VEGF biomarkers have been associated with angiogenesis, inflammation, and bone degradation in the context of RA and therefore serve as reliable indicators for tracking disease progression. Therefore, our findings indicate that FCA administration successfully induced RA in our experimental model, as revealed by the upregulation of inflammatory biomarkers in the FCA-treated model. Next, we explore whether the antirheumatoid activity of TMZ is related to its ability to modulate inflammatory pathways in the RA model. As shown in Figure 5, administration of TMZ or MTX, alone or in combination, substantially diminished (*p* < 0.001) the expression levels of serum IL-6 and VEGF proteins, compared to the FCA-induced RA group. These results indicate the ability of TMZ to mitigate inflammation associated with FCA-induced RA. Our findings also showed that the combination of TMZ and MTX showed the most significant impact on the markers tested (Figure 5). Together, our results indicate that the antirheumatoid activity of TMZ could be linked to its ability to target fibrogenic and inflammatory pathways in RA disease and further highlight the synergistic administration of TMZ and MTX as a potential approach to reduce adverse effects of MTX, while improving therapeutic efficacy.

### 2.8 TMZ significantly attenuated articular cartilage damage, thickening of synovial membrane, synoviocytes, and inflammatory cell infiltration

**FIGURE 6:**
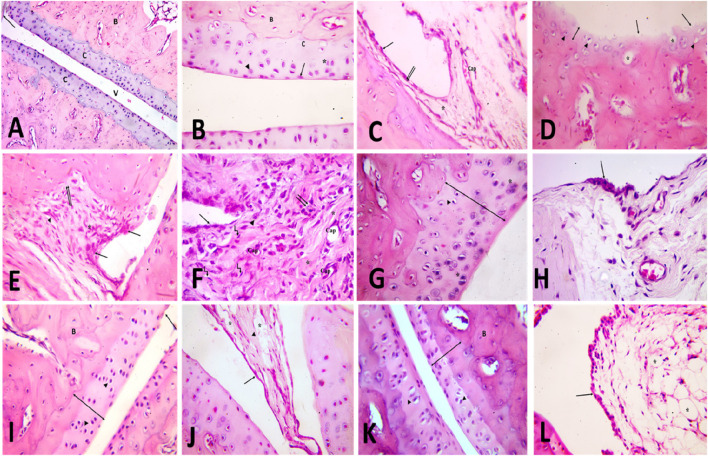
[A, B&C] H&E-stained slices of the ankle joint obtained from the ***control group* (A)** reveal the presence of articulr cartilage **(C)**, the underlying bone **(B)**, the joint cavity (v) and the synovial membrane (). Multiply the given value by 100. [B] the image illustrates the various zones of articular cartilage, namely, the superficial tangential zone (), the transition zone () characterized by dispersed chondrocytes, the radial zone () where chondrocytes are aligned perpendicularly to the surface, and the calcified cartilage zone **(C)**. Calcified cartilage is observed to be located above the subchondral bone. The user has provided a numerical value of x400. [C] The composition of the intimal layer of the synovial membrane consists of two main cell types: type A synoviocytes, which resemble macrophages, and type B synoviocytes (), which resemble fibroblasts. The stroma contains blood capillaries (Cap) and adipocytes ().x400. **(D,E,F)** H&E-stained slices of the ankle joint obtained from the ***rheumatoid arthritis*** (RA) model group: [D] exhibit the presence of articular cartilage characterized by an uneven surface and a noticeable reduction in its formerly smooth contour (). The absence of basophilia inside the cartilage matrix and a noticeable reduction in its thickness (vertical dimension) are observed (). Empty chondrocyte lacunae () and degenerate chondrocytes with pyknotic nuclei () are seen. X400. **(E)** shows the synovial membrane penetrating into the underlying cartilage and bone (). An apparent increase in the number of synoviocytes that line the synovial membrane is observed. Mononuclear cell aggregations () and an apparent increase in collagen fibers are seen in the stroma (). X400. **(F)** shows infiltration of the synovium by inflammatory cells (). It contains many blood capillaries (Cap), with an apparent increase in collagen fibers in the stroma (). Many spindle-shaped cells (kinked arrow) and occasional multinucleated giant cells () are also seen. Notice fibrin deposition () on the cells that line the synovial membrane. X400. [G&H] H&E-stained slices of joints obtained from the ***TMZ group* (G)** exhibit articular cartilage characterized by its basophilic matrix and chondrocytes occupying their lacunae. A limited number of chondrocytes exhibit cell shrinkage and possess nuclei with a darkened appearance (). Notice the apparent increase in cartilage thickness X400. **(H)** the observed findings indicate a modest elevation in the intimal thickness of the synovial membrane. Notice the apparent decrease in inflammatory cells X400. **(I,J)** the H&E-stained slices of the joint obtained from the ***MTX group*: (I)** illustrate the presence of articular cartilage, which consists of four distinct zones, as well as subchondral bone **(B)**. There is an observable increase in cartilage thickness (). Arbitral cartilage appears with a slight irregularity of the surface (), but with a preserved basophilic matrix and chondrocytes within its lacunae (). X400. **(J)** shows the synovial membrane lined with synoviocytes (). Collagen fibers () and adipocytes () are seen in the stroma. X400. [K&L] H&E-stained sections of joints obtained from the ***combination group***: **(K)** demonstrate the presence of the articular cartilage, which exhibits four distinct zones, as well as subchondral bone **(B)**. Observe the evident increase in cartilage thickness () with preserved basophilic matrix and chondrocytes inside their lacunae (). X400. **(L)** Showing the synovial membrane lined with synoviocytes (). Many fat cells () are seen in the stroma. X400.

To further confirm our biochemical results, a detailed histological examination of ankle joint sections was estimated. In the control group, articular fluid filled the cavity of the joint, creating two surfaces of the ankle joint visible under H&E staining. Articular cartilage provided a smooth surface for the bones it covered. Hyaline cartilage, which is articular cartilage, lacks a covering perichondrium. A synovial membrane was found to line the inside of the joint space (Figure 6A). Arsenic cartilage was classified into four discrete layers, commonly referred to as “zones.” The first layer, known as the outermost layer or “tangential” layer, consisted of elongated chondrocytes oriented parallel to the surface. The second layer, known as the second or “transitional” layer, contained scattered chondrocytes that were more round in shape. The third layer, known as the third or “radial” layer, consisted of spherical chondrocytes arranged in columns perpendicular to the surface (Figure 6B). The synovial membrane was found to be composed of a lining of synoviocytes (intima), consisting of two to three layers of epithelium-like cells. There are two different types of synoviocytes that can be observed within the synovial membrane: synoviocyte type A, which exhibits characteristics similar to macrophages, and synoviocyte type B, which resembles fibroblasts (Figure 6C). showed the presence of adipocytes, capillaries, and a limited number of collagen fibers within the stroma, which serves as the underlying layer of connective tissue.

Articular cartilage in the model group for rheumatoid arthritis (rheumatoid arthritis group) was thinner and lost basophilia in H&E-stained slices compared to the control group. Apoptotic chondrocytes predominated, and many of them showed signs of shrinkage and eccentric pyknotic nuclei. Many spots in the articular cartilage revealed cell death and most of the lacunae appeared empty. Articular cartilage lost its smooth contour and developed erosion on its surface, as seen in (Figure 6D). There appeared to be an increase in the number of synoviocytes that lined the synovial membrane, resulting in a thickening of the membrane. Synovial tissue clearly showed invasion by inflammatory cells. The pannus, a protruding portion of the inflamed synovium that contains inflammatory cells and fibroblasts, expanded over the surface of the articular cartilage, eroding the cartilage and bone underneath it (Figure 6E). Inflammatory cells, mononuclear and multinucleated giants, were observed in the synovial stroma. There was also a discernible increase in the number of collagen fibers and blood vessels. The synovium consistently showed hyperplasia of spindle-shaped cells (Figure 6F).

The arthritic joint pathology induced in the TMZ-treated group was improved by injection of trimetazidine. Articular cartilage was found to be significantly thicker in this group than in the RA model group. Articular cartilage filled its crevices with chondrocytes and possessed a smooth basophilic matrix. However, some of the chondrocytes seemed to deteriorate, with their cell bodies shrinking and their nuclei severely stained (Figure 6G). Two to three layers of synoviocytes bordered the synovial membrane (Figure 6H).

The cartilage articular obtained from the MTX treated group exhibited a surface characterized by smoothness, a basophilic stained matrix, and the presence of chondrocytes inside intercellular spaces, as visually demonstrated in (Figure 6I) by staining with hematoxylin and eosin. Compared to the RA model group, the thickness of the articular cartilage increased significantly. The synovial membrane was lined with synoviocytes, and fat cells and blood vessels were found in the connective tissue underneath (Figure 6J).

The management of the combination group almost mirrored that of the control group. The four distinct areas of articular cartilage were clearly seen. (Figure 6K). showed that the thickness of the cartilage has increased significantly compared to the RA model group, with the basophilic matrix and chondrocytes still present within the lacunae. The synovial membrane was lined by a few layers of synoviocytes, and adipose tissue lay beneath them (Figure 6L). These findings validate our biochemical results and demonstrate the antirheumatoid efficacy of TMZ and MTX in the inflamed joint induced by FCA treatment. Furthermore, they contribute to improving the thickness of articular cartilage and its replenishment with chondrocytes, resulting in a smooth basophilic matrix. These results underscore the antirheumatic potential of both drugs, with a particularly favorable effect observed in the combination group.

### 2.9 TMZ significantly increased FADD positive vesicles in the synovial membrane

**FIGURE 7:**
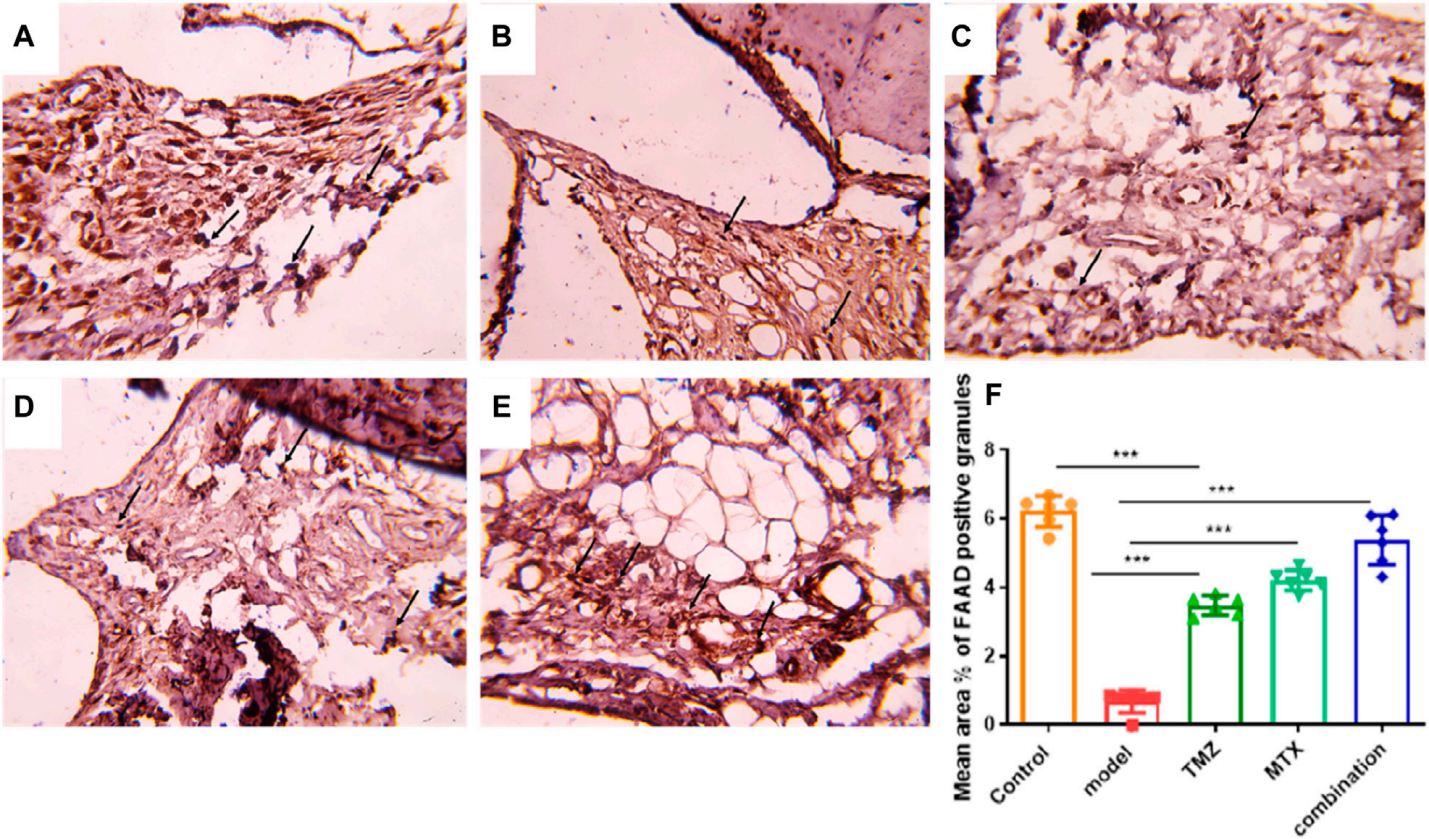
Diaminobenzidine immunohistochemically stained sections for the FADD protein from different groups **(A)** Showing the FADD protein in the synovial membrane by immunohistochemistry Multiple brownish granules () positive for FADD immunostaining are seen. Control group. x 400. **(B)** Showing a few brownish FADD positive granules () in the synovial membrane RA model group, x400 **(C)** Showing multiple brownish granules () positive for FADD immunostaining. TMZ group, x400. **(D)** Showing multiple brownish granules () positive for FADD immunostaining MTX group, x400 **(E)** Showing multiple brownish granules () positive for FADD immunostaining. Combination group, x400. **(F)** Mean area % of FAAD positive granules in the synovial membrane for the different groups. Data are presented as mean ± SE (n = 6). Multiple comparisons were analyzed by one-way ANOVA followed by Tukey’s multiple comparison test. Differences between groups were considered significant when *p* < 0.05 (∗*p* < 0.05; ∗∗*p* < 0.01; ∗∗∗*p* < 0.001).

To gain further evidence for our presented innovative mechanism underlying the antirheumatic properties of TMZ and MTX, the immunohistochemical study of the synovial membrane was accomplished. In the control group, multiple FADD-positive vesicles were observed on the synovial membrane (Figure 7A). Compared to the control group, the synovial membrane of the rheumatoid arthritis (RA) model group showed a significantly reduced abundance of FADD positive vehicles compared to the control group (Figure 7B). Unlike the cohort with rheumatoid arthritis, the cohorts receiving TMZ, MTX and the combination thereof exhibited a substantially higher prevalence of FADD positive vesicles (Figures 7C–F). However, a nonsignificant difference was noticed between the combination group and the control group. These results demonstrate the protective effect of both drugs and their synergetic administration *through* prevention of microvesicular shedding of FADD vesicles, and this effect emphasized our unique pathway and highlighted the role of FADD vesicular shedding in the pathogenesis of rheumatoid arthritis with preferred effect toward the combination group.

### 2.10 TMZ significantly decreased FADD macrovesicles in synovial fluid

**FIGURE 8:**
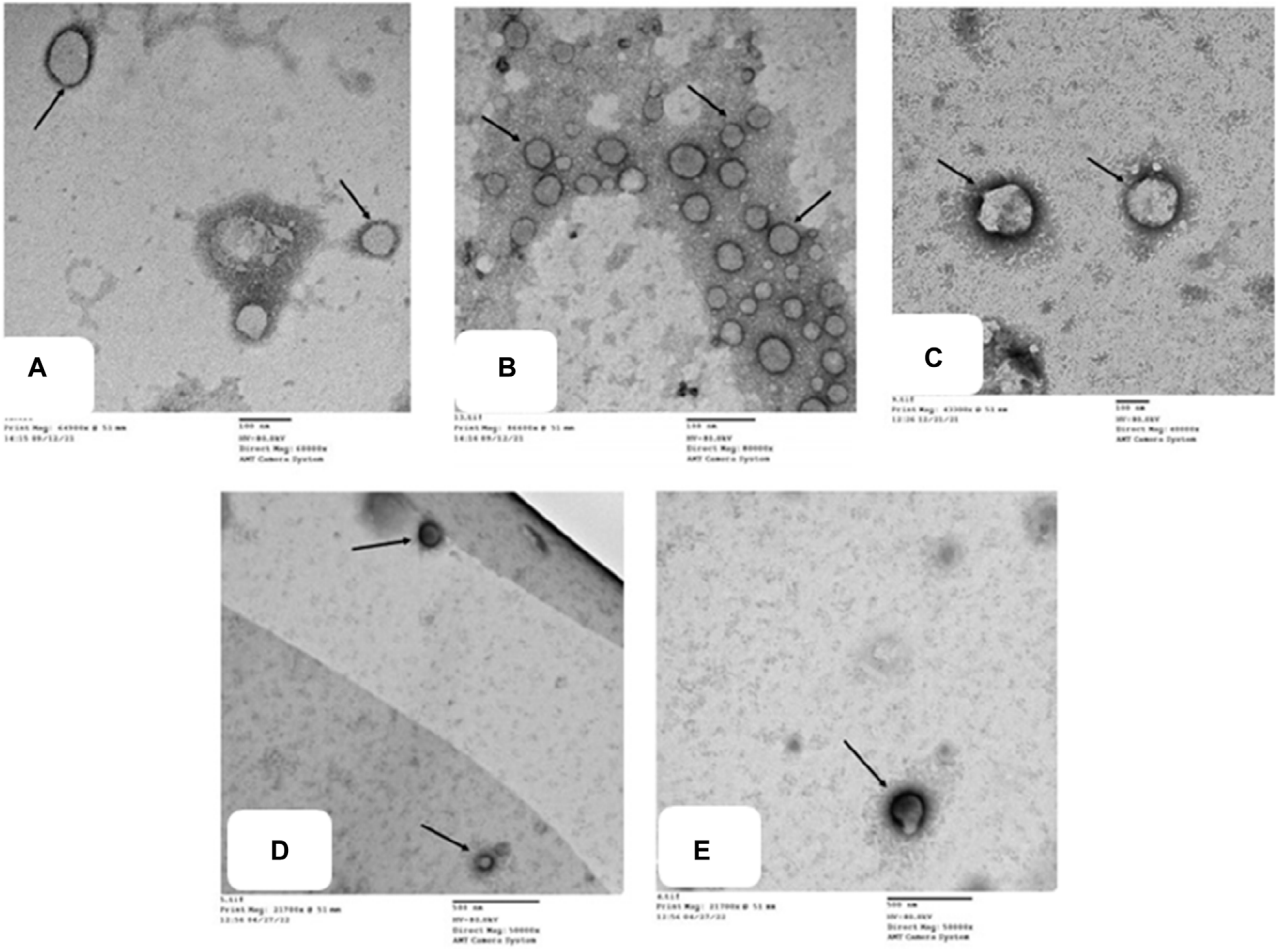
Transmission electron micrographs of different groups **(A)** show microvesicles of different diameters in the SF. Phosphotungstic acid is seen concentrated on the cell membrane of macrovesicles (). Control group, TEM X 60,000. **(B)** shows multiple microvesicles of variable sizes () in the SF. RA model group, TEM X 80,000. **(C,D,E)** show a few microvesicles () in the SF. TMZ group, TEM X 400,000; MTX group, TEM X 50,000; and Combination group, TEM X 50,000.

To confirm our histological results and gain insight into the possible antirheumatoid effect of the drugs examined, we performed detailed TEM analysis for synovial fluid to assess the ultrastructural alterations of the joint FCA-induced RA model and the groups treated with TMZ and MTX. The impact of TMZ and MTX, alone or in combination, on SF of the FCA-induced RA model is presented in Figure 8. The TEM examination of the SF of the control group showed a few macrovesicles of different diameters. Phosphotungstic acid is seen to be concentrated on the cell membrane of macrovesicles (Figure 8A). Although TEM examination of the SF in the FCA-induced RA model group showed an apparent increase in the number of macrovesicles compared to the control group (Figure 7A), Few macrovesicles were seen in the SF of the TMZ, MTX and combination groups (Figures 8C–E). These findings confirm the elevation of the number of microvesicles in the FCA-induced RA model. It is noteworthy that TMZ treatment, alone or in combination with MTX, dramatically reduced FADD microvesicular shedding compared to the FCA treated model group. These findings underscore the validity of our proposed pathway, in which we observed an increase in FADD vesicles in synovial fluid (SF) in the FCA-induced RA group. However, treatment with TMZ and MTX, individually or in combination, resulted in a reduction in the shedding of the FADD vesicle. This reduction is indicative of their capacity to raise adenosine levels, subsequently preventing microvesicular FADD shedding and thus inducing their anti-inflammatory effects through inhibition of NFκB expression. These results collectively provide evidence of the antirheumatic properties of both drugs, with a notable preference for the combination therapy group.

### 2.11 Histomorphometric assessments

Finally, we performed a histomorphometric examination of the thickness of the articular cartilage to corroborate our histological findings. In the rheumatoid arthritis model group, there was a significant decrease in the mean thickness of the articular cartilage compared to the control group (Table 1). In all treated groups (TMZ, MTX, and combination groups), a significant increase in the mean thickness of articular cartilage was observed compared to the RA model group. However, a nonsignificant increase in the mean thickness of the articular cartilage was observed in the combination group compared to the control group (Table 1). These results further substantiate the synergistic antirheumatic efficacy of TMZ and MTX, a phenomenon corroborated by the previously presented biochemical and histological findings.

**TABLE 1:** Thickness of the articular cartilage in different groups (Mean ± SD).

Group	Mean thickness articular cartilage
Control	74.50 ± 2.58
RA model	47.16 ± 2.92^a^
TMZ	59.00 ± 2.36^a^ ▲
MTX	66.16 ± 2.92^a^▲
Combination	72.20 ± 2.28 ▲

^a^ Significant change compared to control; Significant change compared to the RA, group.

### 2.12 Molecular modelling assessments

**FIGURE 9:**
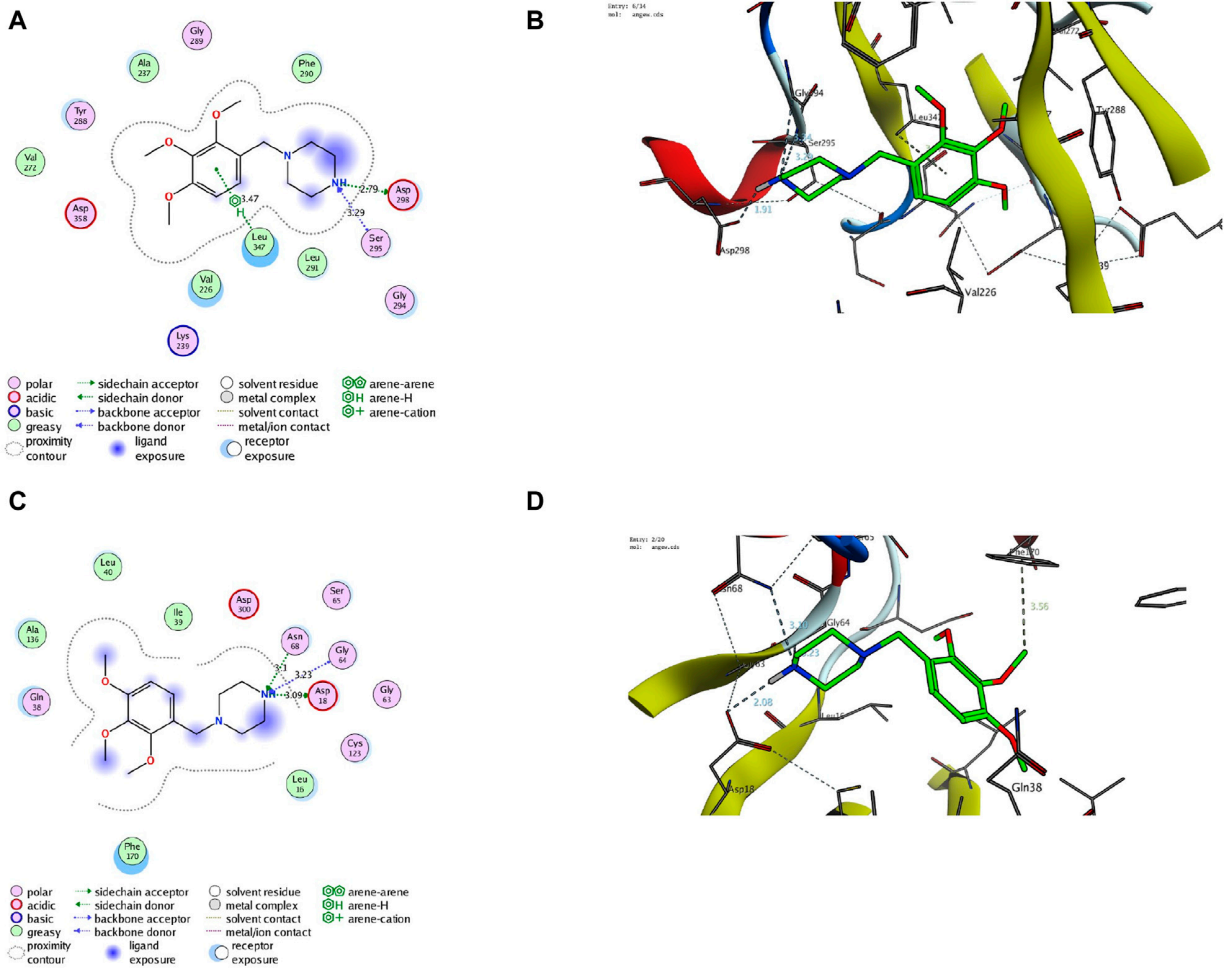
Descriptive 2D and 3D binding modes of TMZ inside the pocket of the IRAK1 protein (PDB code: *6BFN*) **(A,B)** and the adenosine kinase protein (PDB code: *2I6B*) **(C,D)**.

Finally, we aim to affirm the ability of TMZ to target interleukin-1 receptor-associated kinase 1 (IRAK1) and modulate adenosine levels by assessing its binding affinity to IRAK1 and adenosine kinase proteins. In this regard, molecular modeling analysis has previously affirmed the ability to explore the mode and affinity of drugs toward the targeted proteins (Gaber et al., 2020; Samaha et al., 2020; El Azab et al., 2021; Mohamed et al., 2021; Saied et al., 2021; Khirallah et al., 2022a; Mohamed et al., 2022a; Khirallah et al., 2022b; Mohamed et al., 2022b; Healey et al., 2022; Salem et al., 2022; Radwan et al., 2023). In the current study, we have performed detailed *in silico* molecular coupling studies for TMZ toward IRAK1 and adenosine kinase binding sites utilizing the Molecular Operating Environment (MOE) program. Toward this end, we have acquired the crystal structure of IRAK1 (PDB code: *6BFN*) and adenosine kinase proteins (PDB code: *2I6B*) from the reported database (Wang et al., 2022; Muchmore et al., 2022). The modelling protocol was initially adjusted to ensure that the cocrystallized ligands bind to the active sites of the target proteins in a mode similar to that reported with low RMSD values. As shown in Supplementary Figure S2, the cocrystallized ligand of pyridine-2-carboxamide demonstrated a substantial binding affinity to the IRAK1 pocket through mainly two hydrophilic interactions with amino acids Leu291 and Asp358 (2.93 A ° and 2.81A°, respectively). The modeling analysis revealed that the pyridine-2-carboxamide cocrystallized ligand exhibits a low RMSD value and high binding affinity with −17.36 kcal/mol. Furthermore, the cocrystallized ligand interacted with a set of hydrophobic amino acids, including Ile218, Phe223, Val226, Ala237, Val272, Phe290, Pro292, and Leu347. Assessment of the binding mode of TMZ revealed that TMZ has a considerable binding affinity to the active site of the IRAK1 protein (−18.42 kcal/mol). Evaluation of the TMZ binding mode indicated that TMZ has the ability to form hydrogen binding with three amino acid residues (Ser295, Asp298, and Gly294). Furthermore, TMZ demonstrated a substantial H-arene interaction with the Leu347 amino acid (Figure 9). The binding mode of TMZ was further stabilized by forming hydrophobic interactions with Val226, Ala237, Val272, Phe290, and Leu291 amino acids. These results are in agreement with our presented findings that TMZ has a substantial ability to attenuate the activity of the IRAK1 protein, which could be attributed to its ability to bind to the active site of the IRAK1 protein.

Our biochemical analysis revealed that TMZ treatment enhances adenosine levels that could be associated with adenosine kinase activity. To explore this hypothesis, we next assessed the binding affinity of TMZ to the active pocket of the adenosine kinase protein. Toward this end, we first evaluate the modelling protocol by evaluating the binding affinity of the pyrimidin-4-amine acetylinic cocrystallized ligand. As shown in Supplementary Figure S2, the cocrystallized pyrimidin-4-amine acetylinic ligand demonstrated a substantial binding affinity (−11.63 kcal/mol) to the adenosine kinase pocket by forming hydrogen binding with the amino acid residues Leu16 and Ser65 (3.89A ° and 2.87A°, respectively). The binding of the pyrimidin-4-amine acetylinic cocrystallized ligand was further extended to hydrophobically bind to amino acids Ile39, Leu40, Ala136, Leu138, Phe170, and Phe201. On the other hand, TMZ showed the ability to substantially bind to the active site of the adenosine kinase protein with high affinity (−13.57 kcal/mol) by hydrophilically binding to two essential amino acid residues, including Gly64 and Ser65 (3.65A° and 3.71A°, respectively). Furthermore, TMZ exhibited two strong H-arene interactions with amino acids Phe170 and Phe201 (4.62A ° and 3.79A°, respectively) (Figure 8). Analysis of the binding mode also revealed that TMZ forms hydrophobic interactions with the pocket of adenosine kinase (Leu16, Leu40, and Leu138). Taken together, these results indicate that the ability of TMZ to upregulate adenosine levels could be associated with its ability to attenuate the activity of the adenosine kinase protein. Further studies should be directed to explore this hypothesis and affirm the inhibitory activity of TMZ.

## 3 Discussion

Research on rheumatoid arthritis has received more attention in recent years due to the increasing prevalence of RA worldwide and the significant side effects associated with current medications (Bullock et al., 2019). Therefore, the development of potent antirheumatoid drugs is of paramount importance. In the current study, we explore, for the first time, the antirheumatoid effect of TMZ in the FCA-induced RA model. FCA is commonly used to artificially induce arthritis and other autoimmune disorders (Billiau and Matthys, 2001). After a subcutaneous injection of FCA, arthritis in rats manifests in two distinct phases: initial appearance of periarticular inflammation, followed by subsequent bone involvement, reflecting the progression observed in human arthritis cases (Loredo Pérez et al., 2016). In the current investigations, male Wistar rats were treated with FCA to induce arthritis, which resulted in paw inflammation and increased paw volume, but also ankle diameter. These findings were in line with previous reports (Saad et al., 2019; Huang et al., 2020). However, there was a trend towards a marginally smaller body mass in the FCA-treated model compared to the control group. Inflammation could potentially be related to weight loss in individuals with RA, as previously documented (Sun et al., 2021). Aligned with other reported therapeutic agents (Alavala et al., 2020; Huang et al., 2020), the drug-treated groups (TMZ, MTX, and combination groups) showed a notable decrease in paw volume and ankle diameter compared to the FCA model. The nonsignificant decrease observed in body weight of drug-treated groups (TMZ, MTX, and combination groups) might be attributed to the accessibility of food, conveniently located inside the cage, which renders arthritis or its resolution less likely to influence their eating capabilities.

Our biochemical and Western blot analysis indicated that the FCA treated group exhibits a significant attenuation in the expression of FADD compared to the control group. In contrast to our findings, an increase in FADD expression was observed in RA and was interpreted to potentially exhibit a detrimental effect. Importantly, these investigations examined the expression of FADD *in vitro* in various types of immune cells, while our study focused on the entire synovial tissue (Chen et al., 2022). These conflict results may be explained by the measured level of FADD but not the levels of FADD protein, indicating that the expression of FADD mRNA increased in RA but with increased extracellular excretion. These findings were supported by electron microscopy analysis, which revealed an increased number of microvesicular in the FCA-induced RA model. In agreement with our findings, elevated levels of FADD-containing microvesicles have been detected in synovial fluid and blood serum of individuals diagnosed with RA (Jung and Mun, 2018). Furthermore, the FCA model was shown to lead to a reduced tissue level of FADD attributed to its extracellular shedding (Mouasni et al., 2019). Administration of TMZ, on the other hand, resulted in a significant elevation in FADD levels within the tissue. Furthermore, a notable reduction in microvesicular shedding of FADD was observed in the TMZ-treated group compared to the TCA-model group. These particular findings were exclusive to our research study. It should be noted that the effect of TMZ on modulating FADD expression and microvesicular shedding was similar to that of the well-known antirheumatoid MTX drug. Additionally, the combination of both drugs showed the most potent effect, suggesting that TMZ is a dose-saving drug to reduce the adverse effects of MTX (Figure 9). The pivotal role of miR-128a in modulating Fas-mediated apoptosis in human T-cell leukemia by directly targeting FADD led us to further explore their interplay in the realm of RA (Menigatti et al., 2013). While the association of miRNA-128a and FADD has been studied in other diseases (Bartel, 2009), our study provided the first evidence for the possible role of miRNA-128a as a potential epigenetic regulator for the expression of FADD, underscoring its importance as a diagnostic marker in RA. Our study revealed a significant increase in miRNA-128a expression in paw tissue in response to FCA administration compared to the control group, indicating a pronounced influence of FCA on upregulating miRNA-128a. On the contrary, in the drug-treated groups, particularly with TMZ alone or in combination with MTX, there was a significant decrease in miRNA-128a expression compared to the FCA-treated model, with a preference effect towards the combination group. In an interesting study, the role of miR-128a in Fas-mediated apoptosis was examined by using Jurkat cells resistant to the Fas-activating antibody (CH-11) and Jurkat/R cells resistant to the Fas antibody. In line with our findings, this study revealed that miR-128a expression led to Fas resistance by directly targeting FADD. On the contrary, antagonizing miR-128a expression sensitized Fas-resistant Jurkat/R cells to apoptosis, underscoring the regulatory influence of miR-128a on FADD expression (Yamada et al., 2014). The secretion of FADD by microvesicles and the extracellular secretion of FADD were shown to be regulated by adenosine and adenosine receptors (Tourneur et al., 2008). Our study revealed a significant reduction in adenosine levels within the FCA model, which aligns with previous observations in the FCA model of female Wister rats (Arunsi et al., 2022). Furthermore, previous research showed that FCA-treated Sprague-Dawley rats exhibit a lower level of cAMP (an adenosine precursor) (Antwi et al., 2022). This finding corroborated previous research showing that adenosine receptors in MH7A cells have an anti-inflammatory effect (Konishi et al., 2022). We observed a significant increase in adenosine levels after the administration of TMZ and MTX, with the most pronounced increase observed in the combination treatment group. Furthermore, molecular coupling studies indicated that the ability of TMZ to regulate adenosine levels could be related to its ability to target the adenosine kinase protein. These findings indicate that TMZ acts by modulating adenosine levels, which play a vital role in modifying FADD expression and attenuating microvesicular shedding in joint tissues (Figure 9).

Ospelt *et al.* reported compelling evidence supporting the substantial upregulation of TLR-4 in the pathogenesis of RA (Ospelt et al., 2008b). In agreement with this study, we noticed that the expression of TLR4 has been significantly increased in the FCA-treated group, compared to the control group. These findings indicate that modulating the TLR pathway may be an effective strategy to alleviate RA symptoms. Our research demonstrated that both TMZ and MTX, alone or in combination, exerted a notable reduction in TLR4 expression within the paw tissue, with the most favorable results observed in the combined treatment group. These results are in agreement with previous studies that indicated that the administration of nimbolide mitigated arthritis through the downregulation of TLR4 in FCA models (Israr et al., 2023). Furthermore, the daphne cortex and its derivative processed with licorice showed potential antirheumatoid activity by targeting TLR4 expression (Meng et al., 2022). Our study further showed that the FCA-treated model exhibits a significant increase in MYD88 tissue expression. These findings were consistent with those reported by Saad *et al.* (Saad et al., 2019). The expression of MYD88 has also been upregulated in the FCA-treated model in Sprague-Dawley rats (Dong et al., 2017). Our findings are closely aligned with the research conducted by Dong et al. (Dong et al., 2018), which identified an association between elevated levels of TLR4, MYD88, and NFKB in rats treated with FCA. Accordingly, inhibition of the TLR4/MYD88 pathway in fibrocyte-like synoviocytes has been proposed as a promising therapeutic approach for managing RA (Bartok and Firestein, 2010). Despite our research findings, a study did not show statistically significant differences in MYD88 expression between patients and control subjects, suggesting that MYD88 does not play a substantial role in the development of RA (da Silva et al., 2021). On the other hand, Gomes da Silva *et al.* identified upregulation in a specific MYD88 genotype in RA patients, which contributed to increased inflammation in a cohort of 423 RA patients (Gomes da Silva et al., 2021). We showed that TMZ treatment considerably reduced MYD88 expression and that these effects were comparable to those of treatment with MTX; however, the best reducing impact was observed in the combination group (Figure 9). Consistent with our findings, a previous investigation reported that the reduction in protein expression of TLR4 and MYD88 played a pivotal role in mediating the anti-inflammatory effects of the monomer derivative of paeoniflorin (MDP) in FCA-induced RA (Xu et al., 2021). Hydrogen sulfide also demonstrated an antirheumatoid effect by inhibiting an activator of the MYD88 protein (Ding et al., 2023).

Previous studies reported elevated levels of TRAF6 and IRAK, downstream targets of the TLR4/MYD88 pathway, in fibroblast-like synoviocytes, suggesting their role in rheumatogenic processes (Zhang et al., 2022). In line with these findings, we noticed an increase in TRAF6 and IRAK expression in rats treated with FCA, compared to the control group. We showed that administration of TMZ or MTX, alone or in combination, significantly mitigated the expression of TRAF6 and IRAK compared to the FCA treated group. Our analysis demonstrated that the combination of TMZ and MTX led to a substantial reduction in IRAK and TRAF6 levels, which closely resembled that observed in the control group. Furthermore, molecular modelling studies indicated that TMZ has a considerable binding affinity to IRAK active sites. These findings further support the ability of TMZ to modulate the TLR4/MYD88 pathway in the FCA-treated group (Figure 9). Our results aligned with previous reports which showed that the anti-arthritic effectiveness of terpinene-4-ol was attributed to its ability to downregulate IRAK and NFkB protein levels in arthritic models of Sprague-Dawley rats (Aslam et al., 2022). Recently, a newly developed Galangin analog was found to inhibit the LPS-induced inflammatory pathway in RAW264.7 cells in a dose-dependent manner by targeting IRAK mRNA expression (Shin et al., 2023). In another work, the anti-inflammatory potency of Nicorandil was revealed to be associated with its ability to suppress TRAF6 (Saad et al., 2019). In the present study, FCA-induced inflammation resulted in a notable upregulation of the pro-inflammatory factor NFkB, accompanied by a notable downregulation of its inhibitor, IkB. These findings are consistent with a previous investigation conducted on the same model that similarly reported a significant increase in NFkB levels and a concurrent decrease in IKB (Saleem et al., 2020; Ariyo et al., 2022). We also showed that the administration of TMZ or MTX significantly decreased the level of NFkB together with a considerable increase in IkB levels, with the best results produced by the combination of both drugs (Figure 9). These results are consistent with previously reported studies that showed that the antiarthritic effects of various plant extracts, including *Morus mesozygia* leaf, Moringa leaf, difengpienol, plumbagin, Chinese herbal Huayu Qiangshen-Tongbi and Kashayams, are associated with their ability to reduce NFκB levels while increasing IκB expression in an RA rat model (Saleem et al., 2020; Aswathy et al., 2021; Ariyo et al., 2022; Chen et al., 2022; Martin et al., 2022; Shu et al., 2022).

Similarly, our study monitored a considerable increase in serum rheumatological biomarker levels, including ACPA, ESR, CRP, and RF in the FCA-treated model. It was previously established that these biomarkers not only exhibit elevated levels, but also show responsiveness in the context of RA (Sarban et al., 2005; Vander Cruyssen et al., 2005; Benhamou et al., 2010; Kim et al., 2015; Poppelaars et al., 2019; Motta et al., 2023). Changes in ESR serve as a valuable indicator of inflammation in RA and exhibit a direct correlation with the extent of disease severity (Sarban et al., 2005). In fact, RFs are widely observed to be frequently expressed in response to immunizations and subsequent immunological reactions to infections, with the aim of facilitating the elimination of pathogen (Ugolini and Nuti, 2021). According to previous reports, RF synthesis has been observed in B cells and plasma cells that have penetrated the synovium of patients with RA (Kim et al., 1999; Choy and Panayi, 2001). Furthermore, CRP emerges as the most reliable and unbiased metric, serving as a valuable prognostic indicator for the progression of the disease to joint deterioration and functional impairment (Benhamou et al., 2010). Administration of TMZ demonstrated a notable reduction in the expression of serum rheumatological biomarkers, an outcome analogous to that observed with MTX. Interestingly, the combination group showed results that were most closely aligned with the normalization of serum rheumatologic biomarker levels, indicating a potential synergistic resolution effect. In line with these observations, previous reports demonstrated that the anti-inflammatory attributes of Diosmin, Trolox, and Levetiracetam resulted in a decrease in ACPA levels in male Wistar rats with FCA-induced arthritis (Shaaban et al., 2022; Arafat et al., 2023).

**FIGURE 10:**
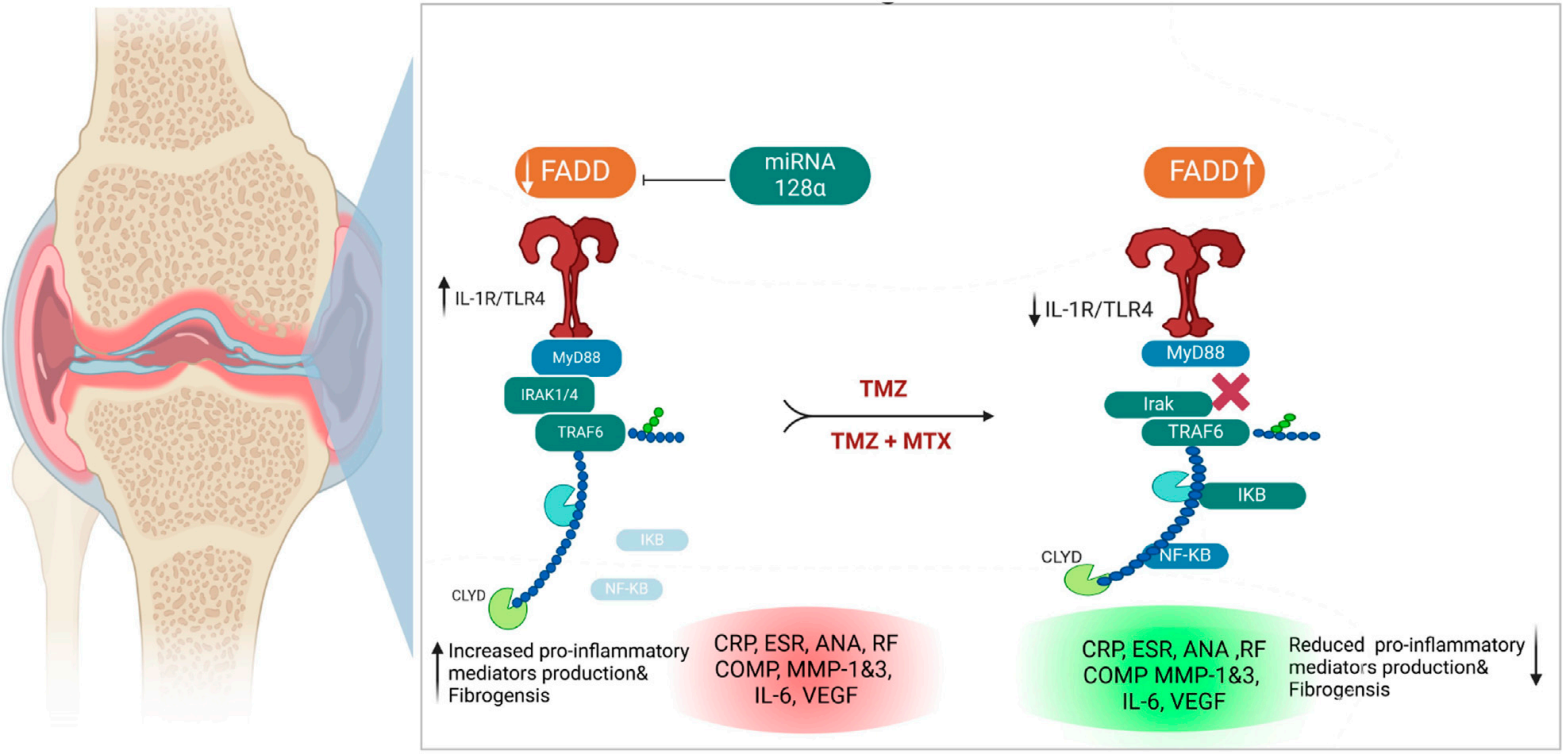
Illustration of the proposed antirheumatoid mode of action of synergetic effect for TMZ and MTX, alone or in combination, in FCA-induced rheumatoid arthritis.

Our investigations extended to the evaluation of blood levels of immunological antibodies, including anti-dsDNA, anti-CarP, anti-MCV, and ANA as potential specific markers for RA. Our results revealed a considerable increase in serum anti-dsDNA, anti-CarP, anti-MCV, and ANA levels in the FCA-induced RA model. Previous research has presented compelling evidence that indicates that individuals diagnosed with RA often exhibit the presence of autoantibodies, including but not limited to anti-dsDNA, anti-CarP, anti-MCV, and ANA (Willemze, 2012; Willemze et al., 2012; Koussiouris and Chandran, 2022). In our study, TMZ treatment demonstrated a notable reduction in serum levels of these antibodies, which was comparable to the effect observed with MTX. In particular, the combination therapy group demonstrated the highest level of efficacy, suggesting a possible synergistic therapeutic effect. The beneficial effects observed in the TMX, MTZ and combination groups may be attributable to their immunomodulatory properties, as evidenced by the reduction in plasma levels of IgG and ANA. The findings of the present study further indicated that the administration of TMX, MTZ, and their combination markedly improved the course of RA, as evidenced by significant reductions in blood levels of serum fibrogenic biomarkers, including COMP, MMP-1, and MMP-3, compared to FCA-induced RA (Figure 9). These findings were confirmed by histopathological analysis of the joint sections. The release of MMPs from activated macrophages during the progression of RA is known to contribute to joint destruction and subsequent release of COMP into the bloodstream. Therefore, suppression of MMP-3 and COMP levels is a clear indication of inhibition of cartilage breakdown (Nogueira et al., 2016). Consistent with these results*,* our research documented a significant increase in serum inflammatory levels of IL-6 and VEGF in the FCA-induced RA model. RA is a chronic inflammatory disease characterized by infiltration of immune cells in the joint synovium, leading to the production of pro-inflammatory cytokines like IL-6 and TNF (Wang et al., 2018). In the synovium affected by RA, VEGF expression increases in macrophages and fibroblasts. Using immunohistochemistry, we have successfully confirmed the presence of VEGF protein expression in endothelial cells inside the synovial tissue (Abdel-Maged et al., 2018). The results of the present investigations suggest that the use of TMX, MTZ, and the combined administration of both drugs significantly mitigated the progression of RA. The efficacy of this intervention was evidenced by notable reductions in circulating levels of IL-6 and VEGF (Figure 10). Aligned with previous histological findings of the FCA-induced model in Wistar rats (Karwasra et al., 2019; Retameiro et al., 2022; Retameiro et al., 2023), we found that the FCA model produced severe joint inflammation with massive infiltration of inflammatory cells, and a hyperplastic synovial membrane that caused erosions in the underlying cartilage and bone with irregularities in surface and pannus development. In line with the histological findings of previous investigations, TMZ treatment reduced the thickness of the synovial membrane, improved the architecture and structure of the cartilage, and smoothed the normal cartilaginous articular surface (Huang et al., 2020; Luo et al., 2020; Patel et al., 2021; Sun et al., 2021; Wang et al., 2023). It should be noted that we are the first to use TMZ as an antiarthritic drug in the FCA-induced RA model. Using immunohistochemistry, we found that TMZ, MTX, and the combination group all increased intracellular FADD, which manifested as brownish spots. These findings add to the evidence that suggests that TMZ may have anti-arthritic properties. Due to its anti-inflammatory properties, TMZ has been shown to have an effect against arthritis. In a rat model of FCA-induced arthritis, the synergistic effect of TMZ and MTX produced the best results by increasing the level of adenosine, which acts through different mechanisms, including inhibition of microvesicular shedding of FADD, eliminating its intracellular anti-inflammatory effect and downregulation of the TLR signaling pathway (Figure 9).

## 4 Materials and methods

### 4.1 Chemicals and drugs

Trimetazidine (Sigma-Chemical Co., Cairo, Egypt) was supplied as a white powder and dissolved in distilled water, while methotrexate was supplied as 50 mg/2 mL vials. Diet, Rat Chow: (20% proteins, 10% 10%fat, 70% carbohydrates) (Animal Food, Egypt) Freund’s complete adjuvant (FCA) (Sigma Aldrich Chemicals, United States. Urethane (Sigma Aldrich Chemicals, United States) was supplied as a white powder and dissolved in normal saline according to body weight at a dose of 1.2 g/kg.

### 4.2 Animals and experimental design

Nile Co. for Pharmaceutical and Chemical Industries, Egypt provided 30 healthy male Wistar albino rats ranging in weight from 150 to 200 g. Rats were kept in a controlled environment with a temperature of 25 ± 3°C and a light/dark cycle every 12 h. They were fed a regular meal and only water to drink. The acclimatization period for rats was 14 days before any procedure. The cages were cleaned well and the bedding was changed daily. This study was authorized by the Research Ethics Committee of the Faculty of Medicine of Ain Shams University (FMASU-REC), and all operations involving animals were conducted in accordance with the National Institutes of Health protocol for the care and use of laboratory animals (NIH Publication No. 85-23, revised 1996). The broad federal assurance number for FMASU-REC is FWA 00006444. After acclimatization for 14 days, the animals were randomly assigned to five groups (n = 6 in each). The five treatments are as follows:

- Group 1 (normal control): During the experiment, rats were injected with 1 mL of 0.9% saline and tap water was given to drink and chewed diet.
- Group 2 (Rheumatoid arthritis model group): On day zero, an experimental model of arthritis in rats was induced by administering a subcutaneous injection of 0.1 mL of FCA to the plantar surface of the right hind paw. On day zero and day one, a 0.1 mL intradermal booster injection was injected into the base of the tail. Due to ethical concerns, FCA was used at a low dose of 1 mg/mL to reduce the number of sick and dead rats. Treatment started the day of arthritis development (the day after injection of the FCA booster dose) and continued for 21 days (Jaleel et al., 2021).
- Group 3 (Trimetazidine; TMZ-treated group): Rats received intraperitoneal trimetazidine (7.2 mg/kg/day) for 21 days (Abdel-Salam and El-Batran, 2005). This dose was chosen based on a pilot study carried out before our experiment and proved to be the best antiarthritic dose.
- Group 4 (Methotrexate; MTX treated group): Rats received intraperitoneal methotrexate (0.75 mg/kg/week) for 21 days (Paulos et al., 2006).
- Group 5 (complementation group): Trimetazidine and Methotrexate treated group (in the same previous doses and duration).

The weights of the animal subjects were recorded at regular intervals of 0, 4, 7, 10, 14, 18, and 21 days.

### 4.3 Evaluation of arthritis

Footpad circumference was recorded at 0, 4, 7, 10, 14, 18, and 21 days (Chen et al., 2023), and measured by measuring the joint’s laterolateral and anteroposterior dimensions, which run perpendicular to one another. A Vernier calliper was used to precisely measure both diameters. The geometric formula for calculating the circumference is as follows:

$$\text{Circumference}=2\pi \;\left[\surd \left(a2+b2/2\right)\right]$$

where a is the laterolateral diameter and b is the antero-posterior diameter (Gomaa et al., 2009).

On the other hand, the Paw volume was recorded at 0, 4, 7, 10, 14, 18, and 21 days and was estimated by placing a liquid column on a scale. The liquid used was distilled water, which was changed each time from the same bottle. An irritated backpaw will experience a force F when submerged in a column of distilled water. Force (F) manifests itself as the weight (W) of the volume of water displaced by the hind paw when it is submerged in the liquid. This force (F=W) can be measured with balance. So, using equilibrium specific gravity of distilled water, we can determine how much of the inflamed hind paw’s volume we are dealing with

$$\text{Mass}/\text{specific}\;\text{gravity}=Volume\left(v\right)$$

Where the specific gravity of water is 1 (Gomaa et al., 2009).

### 4.4 Biochemical assessments

#### 4.4.1 Blood sample collection

The samples were withdrawn from the heart after anesthesia (by intraperitoneal injection of 20 mg/kg of thiopental sodium) and before death, allowed to undergo coagulation for 30 min at 25°C and centrifuged at 3,500 xg for 20 min for serum separation. The serum was then immediately stored at −80°C until use in biochemical analysis.

#### 4.4.2 Anticitrullinated protein antibody (ACPA)

The post-translational conversion of arginine into citrulline results in citrullinated peptides that, in turn, trigger the production of autoantibodies. Early diagnosis of disease has been achieved using serum titration of these autoantibodies (Muller and Radic, 2015). Anti-CCP titers in serum samples were evaluated using ELISA processing equipment (Model Spectra Max Plus-384 Absorbance Microplate Reader, United States) following the manufacturer’s protocols (Cat No. MBS2606863, MyBioSource, San Diego, California, United States). Briefly, plate wells were precoated with rat ACPA and then both the sample and detection antigen were added. After incubation, the plates were washed with PBS (phosphate buffered saline) to remove excess antigens. 3,3′,5,5′-Tetramethylbenzidine (TMB; a coloring substrate) together with its catalytic enzyme (avidin-peroxidase conjugates) were added, and after incubation, another wash was done with PBS. The blue color of TMB turns into yellow by the action of acid (stop solution). The color depth is correlated with the concentration of ACPA in the sample. The optical density was read at 450 nm using a microtiter plate reader in 5 min as described by (Hensvold et al., 2015).

#### 4.4.3 Erythrocyte sedimentation rate (ESR), C-reactive protein (CRP), and serum rheumatoid factor (RF)

The ESR was determined using the Westergren technique. A 3 mL sample of blood was taken by puncturing the retroorbital plexus on the 21st day. This blood sample was then combined with a 3.8% sodium citrate solution in a ratio (4:1, blood: citrate solution). The process of blood mixing was achieved by gently rotating the sample between the palms of the hands. The blood sample was gradually drawn upward until it reached the zero mark within Westergren’s tube. The tube was positioned in an upright manner within the Westergren stand, ensuring that no blood is inadvertently released. The tube was repaired using a screw cap. The measurement of the top level of the red blood cell column was taken after 1 hour and 2 hours. The measurement denotes millimeters of transparent plasma per hour (Ghosh et al., 2018). The CRP measurement was performed using a high-sensitivity latex-enhanced assay, which was provided by Roche Diagnostics, a company based in Almere, Netherlands. The assay was performed on a Hitachi 911 analyzer, also manufactured by Roche Diagnostics, following the instructions provided by the manufacturer. The test has a high level of sensitivity to measuring CRP levels within the range of 0.1–20 mg/L (Asquith et al., 2009). The RF assay was conducted using the latex agaglutination slide test method, employing the RHELAX RF reagent kit from Tulip Diagnostics (P) Ltd. in India. Subsequently, the sera samples were analyzed for the presence of IgM rheumatoid factor using the Hycor Biomedical AutostatTMII Rheumatoid Factor IgM ELISA Kit, located in Garden Grove, California, United States. The tests were carried out according to the manufacturer’s directions (Asquith et al., 2009).

#### 4.4.4 Detection of serum anti-double stranded DNA (anti-dsDNA), anti-carbamylated protein (Anti CarP), antimutated citrullinated vimentin (Anti MCV) and antinuclear antibody (ANA)

Anti-dsDNA levels were estimated using a commercially available ELISA kit manufactured by ORGENTEC Diagnostika GmbH, located in Mainz, Germany. We used a cut-off value of 20 IU/mL, as recommended by the manufacturer and validated in our laboratory. The SLEDAI2000 scores were determined in accordance with the methods outlined in earlier literature (Gladman et al., 2000). The measurement of serum anti-CarP antibody level was conducted using an ELISA technique (Ibrahim et al., 2018). In this study, Nunc MaxiSorp plates (Termo Scientifc) were used. These plates were coated with a solution containing 10 μg/mL of FCS obtained from Bodinco. Additionally, the FCS was carbamylated (Ca) and the coating process was carried out at 4°C for an overnight period. The plates were blocked with a solution containing 1% BSA (Sigma) at 4°C for a duration of 6 h. Subsequently, plates were subjected to an overnight incubation with sera that had been diluted in a 1:50 ratio while kept at a low temperature on ice. The detection of bound antibodies was achieved by incubating the samples for a duration of 4 h at low temperature with horseradish peroxidase-conjugated rabbit anti-human IgG. The visualisation of the bound antibodies was then performed using ABTS. The absorbance measurement was carried out at a wavelength of 415 nm and later converted to arbitrary units (AU) per milliliter using the titration curve of a serum pool derived from more than five samples that tested positive for anti-CarP IgG in the anti-CarP IgG ELISA assay.

Orgentec Diagnostics GmbH’s ELISA kits were used to detect anti-MCV antibodies, and all procedures were performed according to the manufacturer’s recommendations. For this, we used the BEST 2000 ELISA processor. Serum samples were diluted to a ratio of 1: 1,500 and later subjected to incubation in a microtiter plate coated with antigens. This incubation process was carried out at room temperature. In addition, kit standards and controls were included in the incubation process with serum samples. Subsequently, the plates were washed three times to eliminate any protein that was not bound, prior to introducing an anti-human IgG antibody that was coupled with horseradish peroxidase. After the incubation period, the unconjugated conjugate was rinsed out and subsequently the plates were subjected to incubation with the substrate 3,3′,5,5′-tetramethylbenzidine. The color development ceased by introducing acid, after which the optical density of each well was assessed at a wavelength of 450 nm. The analysis was performed using the manufacturer’s cut-off of 20 U/mL unless specified otherwise (Poulsom and Charles, 2008).

Detection of ANA was estimated according to (De Rycke et al., 2003). In this experiment, a diluted serum sample was applied in a 1: 40 ratio in phosphate buffered saline (PBS) onto fixed HEp-2 cells obtained from MeDiCa, Carlsbad, CA. The slides were washed two rounds, each lasting 5 min, using phosphate buffered saline (PBS). Subsequently, a fluorescence labeled conjugate (specifically targeting the heavy and light chains of human IgG, manufactured by MeDiCa) was applied to the slides, which were then incubated for an additional period of 30 min. Following two rounds of washing, a cover-lip was carefully positioned on the slide, and further examination of the slides was conducted using a fluorescence microscope set at a magnification of 40x.

#### 4.4.5 Serum cartilage oligomeric matrix protein (COMP), matrix metalloproteinase (MMP1) and (MMP3)

Blood samples were obtained, followed by separation and quick storage of the sera on ice. The sera samples were rapidly cooled to −20°C in a time frame of 8 h after collection. Subsequently, the samples were moved to a storage facility where they were maintained at −86°C. The quantification of COMP in these samples was performed using an inhibitory enzyme-linked immunosorbent assay, as previously documented with minor adjustments (Clark et al., 1999). In this study, Immulon-4 high-binding 96-well plates manufactured by Dynex Technologies located in Chantilly, VA were utilized. These plates were coated with 50 mL of pure human COMP at a concentration of 2 mg/mL. The coating process was carried out using a plate coating solution consisting of 20 mM sodium carbonate with a pH of 9.2. After the coating step, the plates were left at 25°C for 2 h and subsequently stored at 4°C until they were ready for use. The sera was serially diluted in phosphate buffered saline (PBS)–Tween 20 solution (0.05% Tween 20 in 13 PBS) to achieve final serum dilutions of 1:4, 1:8, 1:16, and 1:32. A volume of 60 μL of diluted sera, together with a series of diluted purified human COMP standard samples (varying from 50 ng/well to 0.78 ng/well in 50 mL), were subjected to incubation with 60 mL of a monoclonal antibody 17-C10 solution diluted at a ratio of 1:20,000. This antibody was specifically developed against human COMP, as previously described (Clark et al., 1999). Overnight incubations at 4°C were performed on a low-binding Microwell plate manufactured by Nunc, located in Naperville, IL. The combination of inhibitors was then placed in the plate covered with COMP and incubated at 4°C for a duration of 1 h. Following the rinsing process, the antibody that had formed a bond was identified using a 1:5,000 dilution of a secondary antibody, specifically an alkaline phosphatase–conjugated goat anti-mouse secondary antibody (Promega, Madison, WI). Quantification of the secondary antibody was performed by incubating it with p-nitrophenyl phosphate (Sigma, St. Louis, MO) in a diethanolamine buffer solution. The diethanolamine buffer was prepared by dissolving 1M diethanolamine and 0.126 mM MgCl_2_ in water, adjusting the pH to 9.8. The incubation was carried out at 37°C. The measurement of the reaction’s optical density was conducted at a wavelength of 405 nm using an Anthos plate reader manufactured by Anthos in Austria. Data were analyzed using Delta Soft II version 3.31 software developed by Biometallics in Princeton, NJ.

Serum levels of MMP-1 and MMP-3 were estimated using an ELISA kit according to the instructions provided by the manufacturer. The methodology used was based on the principles outlined by (Haro et al., 2000). ELISA was used to measure serum levels of MMP-1 and MMP-3. This was carried out according to the manufacturer’s instructions provided by Amersham Pharmacia Biotech, located in Uppsala, Sweden. Serum samples were dilution, with a 1:1 dilution for MMP-1 and a dilution ranging from 1:1 to 1:8 for MMP-3. Then each individual sample was subjected to duplicate assays. The MMP-1 kit exhibited recognition for the complete range of human MMP-1, including free MMP-1 and MMP-1 that is bound to TIMP-1. However, it did not demonstrate recognition for MMP-1 that is bound to α2-macroglobulin. The assay demonstrated a range of 6.25–100 ng/mL, exhibiting a sensitivity of 1.7 ng/mL. The MMP-3 test detected proMMP-3, active MMP-3 and MMP-3/TIMP complexes within the concentration range of 3.75–120 ng/mL. However, it was unable to detect MMP-3 bound by α2-macroglobulin. The assay had a sensitivity of 2.35 ng/mL.

#### 4.4.6 Serum interleukin 6 (IL6) and vascular endothelial growth factor (VEGF)

Specifically, the IL-6-reliant B9 cell line was used. Cells were cultivated using the standard method with one change: the stimulated human mononuclear cell supernatant was replaced with hybridoma growth factor (Cambio, Cambridge, United Kingdom). Microtiter plates with 96 wells and flat bottoms were used for the experiment. In RPMI 1640 medium with antibiotics, 5% heat inactivated foetal calf serum, 5 × 105 M mercaptoethanol, and serial twofold dilutions of heat inactivated test serum, 5000 B9 cells were cultured per well. After incubation for 72 h at 37°C, 5% carbon dioxide, and 50% humidity, proliferation was measured using a spectrophotometric method. For each dilution in the experiment, three separate tests were performed. Using a standard curve established with a reference preparation known to contain 400 IU/mL of IL-6 (Cambio), the IL-6 values (IU/mL) of the test samples were calculated. Our reference preparation had IL-6 activity that was consistent with 91% of the manufacturer’s reported activity compared to the IL-6 standard with reference number 88/514 provided by the National Institute of Biological Standards of the United Kingdom. The amounts of VEGF in the serum samples culture supernatants were quantified using the ELISA technique provided by R&D Systems. The VEGF immunoassay has been specifically developed for the quantification of two isoforms, namely, VEGF165 and VEGF121. The assays and calibrations were performed in duplicate.

#### 4.4.7 Tissue sample collection and homogenization

After scarification of the rat, the right paws were rapidly removed and enclosed in the aluminum sheath in a sample collection tube, then transported to −80°C until the time of biochemical analyzes. After being frozen at −20°C for 12 h to remove any trace of blood, the tissue was homogenized in PBS (10 mg tissue to 100 mL of PBS), weighed, and chopped into minute pieces. Centrifugation of the homogenate at 15000 rpm, at 2°C for 5 min for TLR4, TRAF 6, IRAK, and IKB assay, at 30,000 rpm for 5 min for MYD88 assay, at 3,000 rpm for 20 min for NFKB assay and at 5,000–10000 rpm for 10 min for adenosine assay. The supernatant was collected carefully for assay.

#### 4.4.8 Evaluation of protein expression by an ELISA assay

These assays were based on the quantitative sandwich ELISA technique. An antibody specific for each analyte was precoated onto a microplate; standards and samples were added into the wells and any analyte present was bound by the immobilized antibody. A biotin-conjugated antibody specific to the analyte was added to the wells, and then avidin-conjugated horseradish peroxidase (HRP) was added to the wells. Finally, substrate solution was added to the wells, and color developed in proportion to the amount of analyte bound; therefore, color development was stopped and the intensity of the color was measured. Centrifugation of the homogenate at 15,000 xg, at 2–8° C for 5 min for the FADD, TLR4, TRAF6, IRAK and IKB assays; at 30,000 xg for 5 min for the MYD88 assay; at 3,000 xg for 20 min for the NFKB assay; and at 10,000 xg for 10 min for the adenosine assay. The concentration of each protein was estimated in the collected supernatant by using the sandwich ELISA assay according to the manufacturer (MyBioSource, Southern California, San Diego, United States). FADD was measured using the rat FADD ELISA Kit (Cat.No.PA5-86226, thermoFisher Scientific). TLR4 was estimated using the Rat TLR4 ELISA Kit (Cat.No. CSB-E15822r, CUSABIO, Houston, Texas, United States) at OD 450 nm. MYD88 was evaluated using the MYD88 ELISA Kit (Cat.No. MBS270363196, BIOCOMPARE, San Diego, CA, United States) at OD 450 nm. TRAF6 was evaluated using the TRAF6 ELISA Kit (Cat.No. MBS3808595, Biocompare, San Diego, California, United States) at OD 450 nm. IRAK was estimated using the IRAK ELSA Kit (Cat.No. LS-F1400, LS BIO, Seattle, WA,US) at OD 450 nm. IKB was estimated by using the IKB ELSA Kit (Cat. No. orb567486, Cambridge, United Kingdom), at OD 450 nm. NFKB was estimated using the NFKB ELSA Kit (Cat.No. MBS015549, BIOCOMPARE, San Diego, CA, United States) at OD 450 nm. Adenosine was determined using the rat adenosine ELISA Kit (Cat.No: MBS2606939, Mybiosource, Nanterre, France) at OD 450 nm. To calculate protein concentration, the OD of the samples was compared with the standard curve for each protein.

#### 4.4.9 Assessment of FADD protein expression by Western blot analysis

Western blotting, commonly known as immunoblotting, is a well-known and widely used technique for protein detection and analysis. Electrophoresis of right paw proteins was performed on 10% SDS-PAGE mini gels (Bio-Rad Laboratories, Hercules, CA, United States), followed by standard immunoblotting procedures. Primary antibodies were purchased from Santa Cruz Biotechnology (CA, United States) and treated with membranes overnight at 4°C, as described in (García Cabrerizo and García Fuster, 2019). The membranes were exposed to the secondary antibody the next day. On the same gel, the number of target proteins in each sample was compared with that of the control rats. ECL reagents (Amersham, Buckinghamshire, United Kingdom) were used to detect target protein immunoreactivity and the signal from the attached antibody was captured on an autoradiographic film (Amersham ECL Hyperfilm) after being exposed to the film for 1–60 min. Densitometric scanning (Bio-Rad GS-800 image-calibrated densitometer) was used to quantify autoradiograms.

#### 4.4.10 Assessment of mi-RNA 128a by RT-PCR

Paw tissues were subjected to RNA extraction using Trizol reagent; trizol is a mixture of guanidine thiocyanate and phenol. Subsequently, DNase treatment was performed to ensure the removal of any remaining DNA. To assess mRNA expression levels, isolated total RNA was reverse transcriptionally performed using the PrimeScript^®^ RT reagent kit (TaKaRa, Otsu, Japan). The mir128a response in RT-qPCR was evaluated by synthesizing cDNA from previously extracted total RNA. The reverse transcription process followed the guidelines provided in the TaqMan MicroRNA reverse transcription kit from Symevier Technology (Co., Ltd., Shenzhen, China). The cDNA amplification reaction system consisted of a total volume of 10 μL, comprising 1.0 µL of oligo DT primer, 1.0 µL of dNTP mixture, 2 µg of total RNA, 1 µL of Taq DNA polymerase, and non-ribonuclease distilled water added to 10 µL. The reverse transcription reaction continued at 37°C for 45 min, followed by a denaturation step at 65°C for 5 min. For the subsequent reaction, the system had a total volume of 50 µL. It included 2 µL of cDNA template, 32.5 µL of SYBR-Green Mix (Guangzhou Dongsheng Biotechnology Co., Ltd.), 0.5 µL for the upstream primer and 0.5 µL for the downstream primer. The remaining volume was made up of double distilled water to reach the final 50 µL. PCR amplification involved an initial predenaturation at 95°C for 3 min, followed by denaturation at 95°C for 30 s, annealing at 55°C for 30 s, and extension at 72°C for 60 s. This cycle was repeated 30 times, with a final extension at 72°C for 5 min after the completion of the cycles. The internal control for the reaction was Beta actin. All samples underwent three repetitions and the results were analyzed using the 2^(-ΔΔCq) method. The primer sequences for mir-128a were 5′-ACA​CTC​CAG​CTG​GGT​CAC​AGT​GAA​CCG-3 and 5′-CCC​AAG​CTT​ATG​AAG​CCA​AAT​GAT​GCA​AAA​T-3’. The primer sequences for Beta actin were GCA CCA CAC CTT CTA CAA TG and reverse primer TGC TTG CTG ATC CAC ATC TG.

### 4.5 Histological and immunohistochemical studies

#### 4.5.1 Light microscopic study

After scarification, the ankle joint was covered with skin to preserve its structure and the structure of the paw. The joint was preserved and fixed in 10% formalin filled cups for approximately 2 weeks before processing and block formation. The softening of the samples was done in EDTA to preserve the architecture of the soft tissue for 1 month. Then, they were dehydrated, cleaned and embedded in paraffin to produce 4–6 m thick paraffin sections. Hematoxylin (H) and eosin (E) staining was performed.

#### 4.5.2 Transmission electron microscopy (TEM) of synovial fluid (SF)

The SF sample was taken through the 0.2 mL saline bath slowly and partially injected into the unskinned closed knee by an insulin syringe immediately after sacrifice of the rat without losing any saline amount or injury to the joint coverings wider than the injection site (Barton et al., 2007). The sample was then placed in a sterile empty tube and centrifuged for 5 min at 3,500 xg and then for 10 min at 4,500 xg. The supernatant was then taken to another sterile empty tube immediately for electronic microscopy examination using TEM-JEOL -1,010: 80 KV at the Regional Center for Mycology and Biotechnology (RCMB), AL Azhar University. SF was kept in the refrigerator from the time of aspiration to the time of transport (Szatanek et al., 2015).

#### 4.5.3 Morphometric analysis and immunohistochemistry of the ankle joint

The Department of Histology and Cell Biology of the Faculty of Medicine of Ain Shams University evaluated ankle joint specimens using an image Leica Q Win V.3 application. The microscope, a Leica DM2500 (Leica Microsystems GmbH, Ernst-Leitz-StraBe, Wetzlar, Germany), was linked to the computer via a built-in camera. From each specimen, five different, nonoverlapping fields were captured. Five separate measurements were taken from each photographed item, and an average was then determined. An impartial observer, unaware of the specifics of the specimens being evaluated, took all necessary measurements. The thickness of the articular cartilage was assessed on average. All readings were obtained using high magnification (x400) instruments. Immunohistochemistry by anti-FADD antibodies guided by the technique mentioned in (Bancroft and Gamble, 2008) and just modified according to the manufacturer’s guide (Product # PA5-86226).

### 4.6 Molecular modeling studies

The binding affinity of TMZ to the active pocket of IRAK1 and adenosine kinase proteins was evaluated by performing a molecular modelling study utilizing the MOE program. The structure of the proteins examined was obtained from the protein database (PDB codes: *2I6B*, adenosine kinase; and *6BFN*, IRAK1 protein, accessed on 01.07.2023) (Wang et al., 2022; Muchmore et al.). After acquiring the 2D structure of TMZ using the Chemdraw builder program, the structure was further adjusted to minimum energy and protonated using the MOE program. The structure of the examined proteins was also subjected to deprotonation and deletion of water molecules and extra chains. The protocol applied in the current study was initially adjusted by redocking the cocrystallized ligand and by comparing the obtained interactions to the reported data. In our investigations, we applied the Triangle Matcher placement procedure for the docking process, and the furnished binding affinity was scored utilizing the London dG scoring protocol. The acquired data were assessed and analyzed to finally provide the mode of interaction of the TMZ drug inside the active site of the examined proteins and assessment of its binding affinity.

### 4.7 Statistical analysis

For statistical analysis, the 2018 versions of Microsoft Excel 2010 and GraphPad Prism 8.0.1 were used. Statistical differences between groups were found using a one-way analysis of variance (ANOVA) followed by a *post hoc* “sidac multiple comparison test” for tests between more than two groups. The same group’s paw volume, ankle diameter, and body weight were all recorded on different days. A two-way ANOVA and a *post hoc* “Tukey multiple comparison test” were then used to compare the different groups. A *p*-value of less than 0.05 means that the finding was statistically significant. All results were given as mean ± SD.

## 5 Conclusion

Existing RA treatments, although effective to some extent, are accompanied by unwanted side effects. This study delved into the intricate landscape of rheumatoid arthritis and provided exclusive insights into the role of miRNA128a, the TLR4 signaling pathway and adenosine in RA pathogenesis by modulating FADD tissue expression and FADD microvesicular shedding in synovial fluid. Upregulation of mi RNA128a, TLR4, MYD88, TRAF6, IRAK, and NF-kB, along with downregulation of IkB, FADD, and adenosine levels, underscored the complex network driving RA pathology. Importantly, we observed a negative correlation between miRNA 128a and tissue FADD expression, reinforcing the first evidence for the involvement of mi-RNA 128a in the progression of RA. Our study further demonstrated that TMZ has a potential anti-rheumatoid activity, similar to that of the well-known MTX drug, by targeting the miRNA 128a, TLR4/MYD88, IKB/NF-κB, and adenosine pathways in the RA model. Its efficacy in mitigating the debilitating consequences of RA was affirmed by significant improvements in the physical parameters and histopathological characteristics of arthritic joints, but also by substantial attenuation in the expression of fibrogenic, inflammatory, immunological, and rheumatological diagnostic markers. In particular, combination therapy exhibited the most pronounced effects, highlighting the potential synergy between TMZ and MTX. Taken together, our study underscores the critical role of mi RNA128a as an epigenetic regulator of FADD expression in RA pathogenesis and further suggests that TMZ holds promise as an antirheumatoid candidate, offering improved therapeutic results with a reduced risk of adverse effects. Synergistic administration of TMZ and MTX emerges as an innovative approach to introduce a safer RA therapy and a proper drug combination to save the dose of MTX, thus decreasing its serious adverse effect. These findings could provide hope for more effective and safer treatments for this debilitating autoimmune disease and pave the way for future research that aims to refine and optimize these therapeutic strategies for the benefit of RA patients worldwide.

## Data Availability

The original contributions presented in the study are included in the article/Supplementary Material, further inquiries can be directed to the corresponding author.
