# Supplementary material for: Deciphering the therapeutic potential of trimetazidine in rheumatoid arthritis via targeting mi-RNA128a, TLR4 signaling pathway, and adenosine-induced FADD-microvesicular shedding: In vivo and in silico study
Source: Front Pharmacol. 2024 Jun 11;15:1406939. doi: 10.3389/fphar.2024.1406939 (PMC11196411; doi:10.3389/fphar.2024.1406939)
Supplement: Supplementary file 1 [file DataSheet1.PDF]

# Supporting Information

## Deciphering the Therapeutic Potential of Trimetazidine in Rheumatoid Arthritis *via* targeting mi-RNA128a, TLR4 signaling pathway, and Adenosine-induced FADD-microvesicular shedding: *in vivo* and *in silico* study

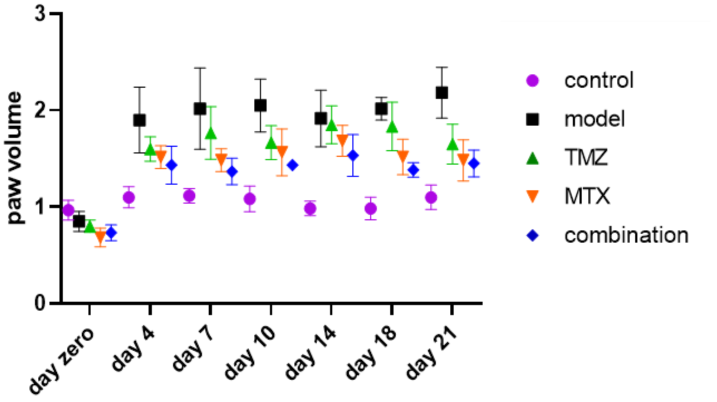


**(A)**

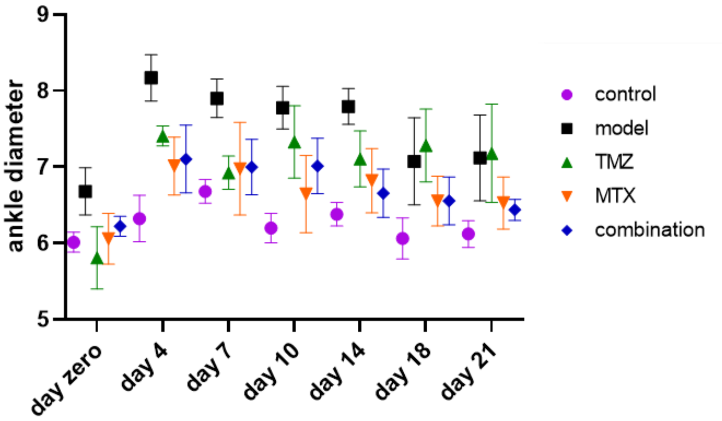


**(B)**

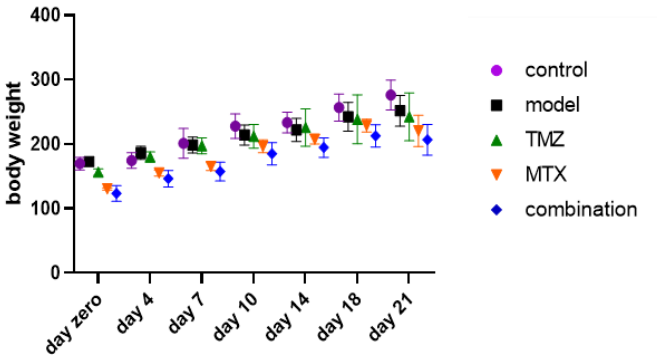


**(C)**

**Figure S1:** The effect of trimetazidine (TMZ), methotrexate (MTX), and their combination on body paw volume (A), ankle diameter (B), and body weight (C) of FCA-induced rheumatoid arthritis (RA) rats as compared to control rats at different times. Data are presented as mean ± SD. Model group (n = 6).

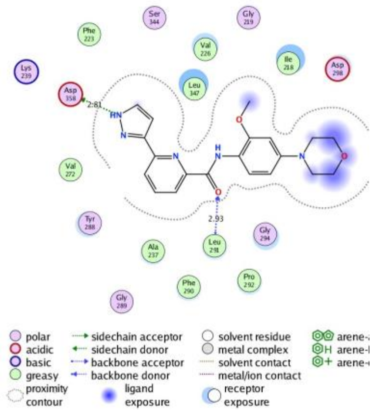


**(A)**

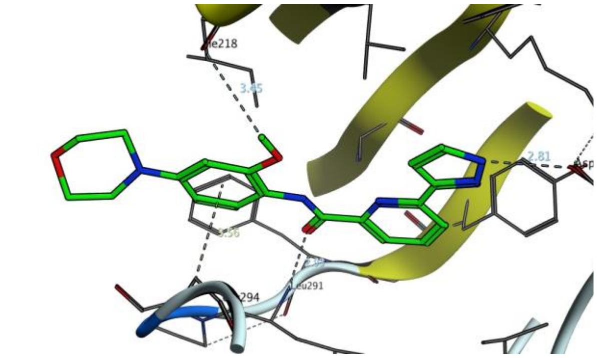


**(B)**

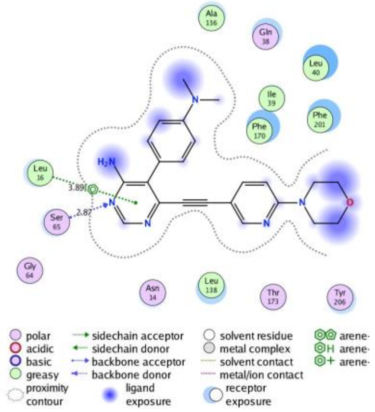


**(C)**

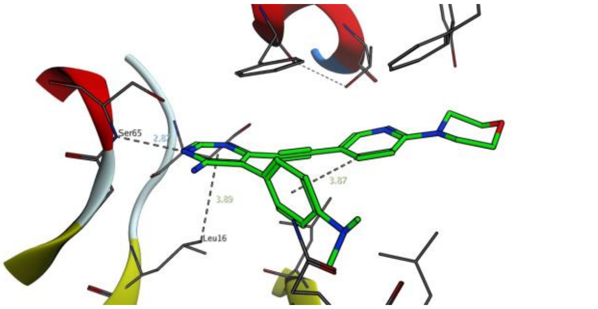


**(D)**

**Figure S2.** (**A, B**) Descriptive 2D and 3D binding modes of the pyridine-2-carboxamide-cocrystallized ligand inside the pocket of IRAK1 protein (PDB code: *6BFN*). (**C, D**) Descriptive 2D and 3D binding modes of the pyrimidin-4-amine acetylinic cocrystallized ligand inside the pocket of adenosine kinase protein (PDB code: *2I6B*).
